# Determining the Relationship Between Blood Pressure, Kidney Function, and Chronic Kidney Disease: Insights From Genetic Epidemiology

**DOI:** 10.1161/HYPERTENSIONAHA.122.19354

**Published:** 2022-09-09

**Authors:** Natalie Staplin, William G. Herrington, Federico Murgia, Maysson Ibrahim, Katherine R. Bull, Parminder K. Judge, Sarah Y.A. Ng, Michael Turner, Doreen Zhu, Jonathan Emberson, Martin J. Landray, Colin Baigent, Richard Haynes, Jemma C. Hopewell

**Affiliations:** Medical Research Council Population Health Research Unit at the University of Oxford, Nuffield Department of Population Health (NDPH), United Kingdom (N.S., W.G.H., S.Y.A.N., M.T., D.Z., J.E., M.J.L., C.B., R.H., J.C.H.).; Clinical Trial Service Unit and Epidemiological Studies Unit, NDPH (N.S., W.G.H., F.M., M.I., P.J., S.Y.A.N., M.T., D.Z., J.E., M.J.L., C.B., R.H., J.C.H.), University of Oxford, Oxford, United Kingdom.; Big Data Institute, Li Ka Shing Centre for Health Information and Discovery (N.S., F.M., M.I., J.E., M.J.L., J.C.H.), University of Oxford, Oxford, United Kingdom.; Health Data Research UK (W.G.H., M.J.L.), University of Oxford, Oxford, United Kingdom.; Nuffield Department of Medicine (K.R.B.), University of Oxford, Oxford, United Kingdom.; National Institute for Health Research Oxford Biomedical Research Centre (M.J.L.), University of Oxford, Oxford, United Kingdom.; Oxford Kidney Unit, Churchill Hospital, Oxford, United Kingdom (W.G.H., K.R.B., P.K.J., M.T., D.Z., R.H.).

**Keywords:** chronic, creatinine, blood pressure, epidemiology, renal insufficiency

## Abstract

It is well established that decreased kidney function can increase blood pressure (BP), but it is unproven whether moderately elevated BP causes chronic kidney disease (CKD) or glomerular hyperfiltration.

**Methods:**

311 119 White British UK Biobank participants were included in logistic regression analyses to estimate the odds of CKD (defined as long-term kidney replacement therapy, estimated glomerular filtration rate [eGFR]< 60mL/min/1.73m^2^, or urinary albumin:creatinine ratio ≥3 mg/mmol) associated with higher genetically predicted BP using genetic risk scores comprising 219 systolic and 223 diastolic BP loci. Analyses estimating associations with clinical categories of eGFR and urinary albumin:creatinine ratio were also conducted, with an eGFR ≥120 mL (min·1.73m^2^) considered evidence of glomerular hyperfiltration.

**Results:**

21 623 participants had CKD: 7781 with reduced eGFR and 15 500 with albuminuria. 1828 participants had an eGFR ≥120 mL/min/1.73m^2^. Each genetically predicted 10 mmHg higher systolic BP and 5 mmHg higher diastolic BP were associated with a 37% (95% CI, 1.29–1.45) and 19% (1.14–1.25) higher odds of CKD, respectively. Associations were evident for both the reduced eGFR and albuminuria components of the CKD outcome. The odds of hyperfiltration (versus an eGFR ≥60 and <90 mL/min/1.73m^2^ were 49% higher (95% CI, 1.21–1.84) for each genetically predicted 10 mmHg higher systolic BP. Associations with CKD and hyperfiltration were similar irrespective of preexisting diabetes, vascular disease, or different levels of adiposity.

**Conclusions:**

In this general population, genetic epidemiological evidence supports a causal role of life-long differences in BP for decreased kidney function, glomerular hyperfiltration, and albuminuria. Physiological autoregulation may not afford complete renal protection against the moderate BP elevations.

**Novelty and Relevance**

**What Is New?**

Previously genetic studies using UK Biobank data have not identified significant associations between genetically predicted blood pressure and estimated glomerular filtration rate. However, once nonlinear nature of associations is taken into account, life-long moderate differences in genetically predicted systolic blood pressure associate with higher risk of both chronic kidney disease (ie, abnormally decreased estimated glomerular filtration rate or albuminuria), and also with glomerular hyperfiltration (ie, abnormally increased estimated glomerular filtration rate). These associations are similar in size in people with or without diabetes, obesity, or vascular disease.

**What Is Relevant?**

Accelerated-phase hypertension is a recognized cause of chronic kidney disease and acute kidney injury, but renal blood flow autoregulation is considered to protect kidneys from moderate hypertension. The presented findings challenge this notion and strengthen claims for a causal link between life-long moderately elevated blood pressure and risk of developing chronic kidney disease.

**Clinical/Pathophysiological Implications?**

Renal blood flow autoregulation may not fully protect kidneys from the effects of moderately elevated systolic blood pressure even in apparently healthy adults. Considering early active management of moderate systolic hypertension in all adults could help reduce individuals’ risk of developing chronic kidney disease in later life.

Conventional observational analyses find higher blood pressure (BP) is associated with chronic kidney disease (CKD) progression^1^ and risk of developing end-stage kidney disease (known as kidney failure).^2,3^ The associations are apparent even among those with only moderate elevations in systolic BP to high-normal levels (ie, >130 mmHg).^3^ However, no clear overall benefit on kidney outcomes emerged from meta-analyses of intensive versus standard BP lowering trials which tested an average BP difference of about 7 mmHg (down to on average about 130 mmHg),^4,5^ raising doubts about whether moderate elevations in BP (in the absence of accelerated-phase hypertension) are an important cause of CKD.

**See editorial, pp 2682–2684**

One potential explanation for the apparent discrepancy between findings from conventional cohorts versus randomized trials is reverse causality. Early kidney disease may be undetected and increase BP^6,7^ resulting in spuriously strong BP-CKD observational associations. A second potential explanation is that intensive BP lowering trials may not have been large or long enough to confirm modest benefits of the achieved BP differences on CKD progression risk.^4,5,8^ Thirdly, it has been suggested that moderate elevations of BP may only cause CKD in individuals with certain comorbid diseases. For example, in healthy individuals, physiological autoregulation of renal blood flow at the glomerular afferent arteriole is considered to protect the kidneys from moderate fluctuations in BP by maintaining a steady filtration pressure,^9,10^ whereas dysregulated renal blood flow homeostasis which predisposes to the development of glomerular hyperfiltration—has been described in people with preexisting diabetes, vascular disease,^10,11^ and obesity.^12^ Post hoc subgroup analyses of intensive BP lowering trials are consistent with such a concept, having hypothesized that benefits of intensive BP lowering may be evident in people with preexisting proteinuria (a marker of dysregulated glomerular function), but not in those without.^13^

Genetic variants are allocated randomly at conception and can be used to proxy an exposure, such as BP, in observational epidemiological analyses, thereby avoiding some of the limitations in conventional observational analyses, such as uncontrolled confounding and reverse causality.^14^ This Mendelian Randomization (MR) approach,^15,16^ has been used to show that moderate life-long genetically predicted differences in BP are associated with risk of myocardial infarction and stroke,^17,18^ replicating the well-established causal relationships confirmed by randomized trials of antihypertensive drugs.^5^ MR has a particular advantage in renal epidemiology as it may help to determine whether the relationship between BP and CKD is bidirectional.^19^ MR evidence supports the existence of causal associations between decreased kidney function and hypertension,^7^ and conversely, between moderate elevations in BP and risk of albuminuria.^17,20^ However, the largest MR studies have found no evidence of association between genetically predicted higher BP and decreased kidney function in adulthood.^7,17^

Previously published MR experiments using UK Biobank data have not identified significant associations between genetically predicted BP and estimated glomerular filtration rate (eGFR).^7,21^ However, these MR studies did not consider that the shape of any associations may be nonlinear.^7,17^ The natural time course of CKD may start with an abnormal increase in kidney function before a subsequent decline in kidney function. Consequently, if genetically predicted BP-eGFR associations are U-shaped (ie, higher BP causes both decreased kidney function and—in other individuals or earlier in the natural time course of CKD—induces glomerular hyperfiltration), analyses using eGFR as a continuous outcome may miss important associations. We aimed to address this deficiency by performing analyses using outcomes based on a categorical definition of CKD used in previously published MR studies (ie, long-term kidney replacement therapy, eGFR<60 mL (mL/min/1.73m^2^), or urinary albumin:creatinine ratio [uACR] ≥3 mg/mmol),^22^ and secondarily, using separate clinical categories of eGFR and albuminuria, with an eGFR ≥120 mL (mL/min/1.73m^2^) considered evidence of glomerular hyperfiltration.

## Methods

### Data Availability Statement

Data supporting this article are available from UK Biobank (http://www.ukbiobank.ac.uk) in accordance with their published data access procedures. Summary data from various genetic consortia as referenced are publically available. All other data are within the article and its Supplemental Material.

### Study Population

UK Biobank is a large prospective cohort study of 502 650 middle-aged adults aged 40 to 69 years recruited between 2006 and 2010 in 22 assessment centers across the United Kingdom. Data include self-completed touch-screen questionnaires, computer-assisted interviews, physical and functional measurements, biochemical assays, and genome-wide genotyping.^23^ At recruitment, seated BP was measured twice using an Omron HEM-7015IT digital monitor, with readings automatically recorded into the computer-based systems. A manual sphygmomanometer was used if the automated device failed to provide a reading. A repeat assessment was conducted among a subsample of ≈5% of the participants from 2012 to 2013. Detailed descriptions of UK Biobank are provided elsewhere.^21^ After exclusions, 311 137 unrelated White British participants were included in all analyses (genetic and observational). The following exclusions were used for all analyses: those who withdrew their data (n=157); those with missing genotype data (n=15 546); those with missing values of BP, age, sex, body mass index (BMI), uACR, or eGFR (n=38 385); non-White British participants (n=71 281) and related individuals (n=65 940).

### Measured BP

Measured BP was calculated as the mean of the 2 measurements taken at recruitment. Participants on antihypertensive medications at recruitment had 15 and 10 mmHg added to the measured systolic BP and diastolic BP values, respectively (as in previous genetic studies^18^).

### Genetic Risk Scores for Systolic BP and Diastolic BP

For genetic analyses, instruments for systolic and diastolic BP and the associated weights were identified from a published genome-wide association studies which combined data from multiple studies, including UK Biobank.^18^ Based on the BP trait most strongly associated with each variant, 219 single nucleotide polymorphism (explaining 1.6% of the systolic BP variance) and a different 223 SNPs (explaining 2.1% of the diastolic BP variance) were selected for respective genetic scores (Tables S1 and S2). Separate genetic risk scores (GRSs) for systolic and diastolic BP were calculated for each participant, based on the weighted sum of the SNP dosages (with weights taken from an International Consortium for Blood Pressure meta-analysis excluding UK Biobank).^18^

### Kidney Outcomes

The primary outcome (referred to as CKD) was a composite defined as long-term kidney replacement therapy, or the 2009 CKD epidemiology collaboration eGFR^24^ calculated from both serum cystatin C and creatinine (eGFR_cys-cr_) <60 mL (mL/min/1.73m^2^), or spot uACR ≥3 mg/mmol. Since the composite outcome contains very different clinical outcomes and because the relationship between kidney disease and GFR is nonlinear in the early stages, in secondary analyses, eGFR- and uACR-based outcomes were analyzed separately based on clinical cutoffs.^25^ For eGFR these were: on long-term kidney replacement therapy or eGFR <45; ≥45,<60; ≥60,<90; ≥90,<120, and ≥120 mL (mL/min/1.73m^2^), with an eGFR ≥120 mL (mL/min/1.73m^2^) considered evidence of glomerular hyperfiltration. For uACR, these were: <3; ≥3,<30; and ≥30 mg/mmol. Lastly, analyses on the effect of hospitalization for acute kidney injury (AKI) reported after recruitment were also performed using the *International Classification of Diseases, Tenth Revision* code N17 (which has high positive predictive value^26^) from any diagnostic position in linked hospital admission records.

### Statistical Analyses

Baseline characteristics (including measured BP) by fifths of each BP GRS are presented. The associations between genetically predicted 10 mmHg higher systolic BP and 5 mmHg higher diastolic BP and the primary outcome of CKD (and separately AKI) were estimated using logistic regression with adjustment for age, age,^2^ sex, BMI (comparable to those covariates included in the International Consortium for BP data used to weight the instrument), top 18 principal components and the array used. For secondary analyses, multinomial logistic regression was used to estimate associations between genetically predicted 10 mmHg higher systolic BP and 5 mmHg higher diastolic BP and odds of each eGFR category versus eGFR ≥60 to <90 mL (mL/min/1.73m^2^) (and, separately, each uACR category versus <3 mg/mmol).

### MR Sensitivity Analyses

MR was also performed with further adjustment for the genetic effects of the BP-related SNPs on type 2 diabetes, BMI, and waist-to-hip ratio (WHR), to assess the direct effects of the BP GRSs on kidney outcomes (ie, not due to indirect effects on diabetes or adiposity). Weights for these genetic effects were taken from publicly available summary data based on individuals of European ancestry in the DIAGRAM (Diabetes Genetics Replication and Meta-Analysis) consortium,^27^ and the GIANT (Genetic Investigation of Anthropometric Trait) consortium meta-analysis,^28^ respectively. For the AKI outcome, analyses additionally adjusted for baseline eGFR and number of hospitalizations were also performed.

Genetic analyses stratified by age, sex, history of diabetes, history of vascular disease, and BMI and WHR (with an interaction term fitted between the BP GRS and the relevant characteristic) explored whether BP associations with CKD, glomerular hyperfiltration, and AKI varied by characteristics which may predispose to dysregulated renal blood flow homeostasis.^10-12^ Stratifying on these characteristics could introduce collider bias if the characteristics are on the causal pathway between the GRS and kidney outcomes. Therefore, sensitivity analyses were conducted stratifying by residual characteristics, defined as the participant’s value of the characteristic minus the genetic contribution to the characteristic from the BP GRS.^29^

Analyses excluding SNPs that could potentially have direct effects on the kidney not mediated through BP were also performed.^30^ SNPs were excluded if they were in (or close to) genes with differential expression in the kidney (see Supplemental Methods– for details of functional annotations and tissue specificity enrichment analysis^31-33^) or have been previously linked to the renin-angiotensin system, TGF (transforming growth factor)-beta, and its signaling pathways, or disordered kidney development/ morphology/ physiology.^18,34-37^ SNPs that explained more variation in kidney function than BP when applying Steiger filtering^38^ were also excluded. See Table S1 for the lists of SNPs excluded in this sensitivity analysis.

The robustness of the MR results to violations of the instrumental variable assumptions, particularly the assumption of no pleiotropic effects, were also explored using standard approaches based on summary data.^39^ MR-Egger provides a robust estimate of the association in the presence of directional pleiotropy (assuming the pleiotropic effects are independent of instrument strength),^40^ while the weighted median approach gives a robust estimate as long as at least 50% of the weight in the analyses comes from variants with no pleiotropic effects.^41^ Approaches that remove variants with heterogeneous estimates (which could suggest potential pleiotropic effects) were also applied, with outliers identified using a modified Q statistic^42^ or MR-PRESSO (Mendelian Randomization Pleiotropy RESidual Sum and Outlier).^43^

### Conventional Cross-Sectional Observational Analyses

Conventional cross-sectional observational associations using measured BP were also estimated to compare with the genetic associations. Potential confounders were identified at baseline and based on the assumed pathways between BP and CKD (the primary outcome) and included age; sex; region; education (college/university degree, A levels/AS levels or equivalent, O levels or equivalent, none of the above, prefer not to answer); Townsend index of social deprivation (fifths); smoking (current smoker versus not); alcohol use (daily, weekly, occasional, never, prefer not to answer); physical activity (<10 metabolic equivalents-h/wk, ≥10–<50 metabolic equivalents-h/wk, ≥50 metabolic equivalent-h/wk); history of diabetes (yes versus no, defined as self-reported, doctor-diagnosed or HbA1c ≥6.5%), and body mass index (BMI [fifths]).

For the conventional cross-sectional observational analyses, binary and multinomial logistic regression adjusted for the potential confounders listed above were used (with additional adjustment for baseline eGFR and number of hospitalizations for AKI). These models included a standard adjustment for regression-dilution bias^44^ to account for any measurement error and short-term variability in BP (using regression-dilution ratios of 0.60 and 0.53 for systolic and diastolic BP, respectively, as estimated from repeated BP measurements at resurvey).

Analyses were performed in SAS version 9.4 (SAS Institute, Cary NY) and R v3.6.2.

## Results

### Population Characteristics

Among the 311 119 participants included in analyses, mean (standard deviation) age was 57 (8) years, 144 667 (46%) were men, and mean (standard deviation) BMI was 27.4 (4.7) kg/m^2^. 16 282 (5.2%) and 18 168 (5.8%) reported a history of preexisting diabetes or vascular disease, respectively (Table), with 71 784 (23.1%) prescribed antihypertensive medication. Mean (standard deviation) systolic BP was 141.7 (20.6), and diastolic BP was 84.6 (11.2) mmHg.

**Table.**
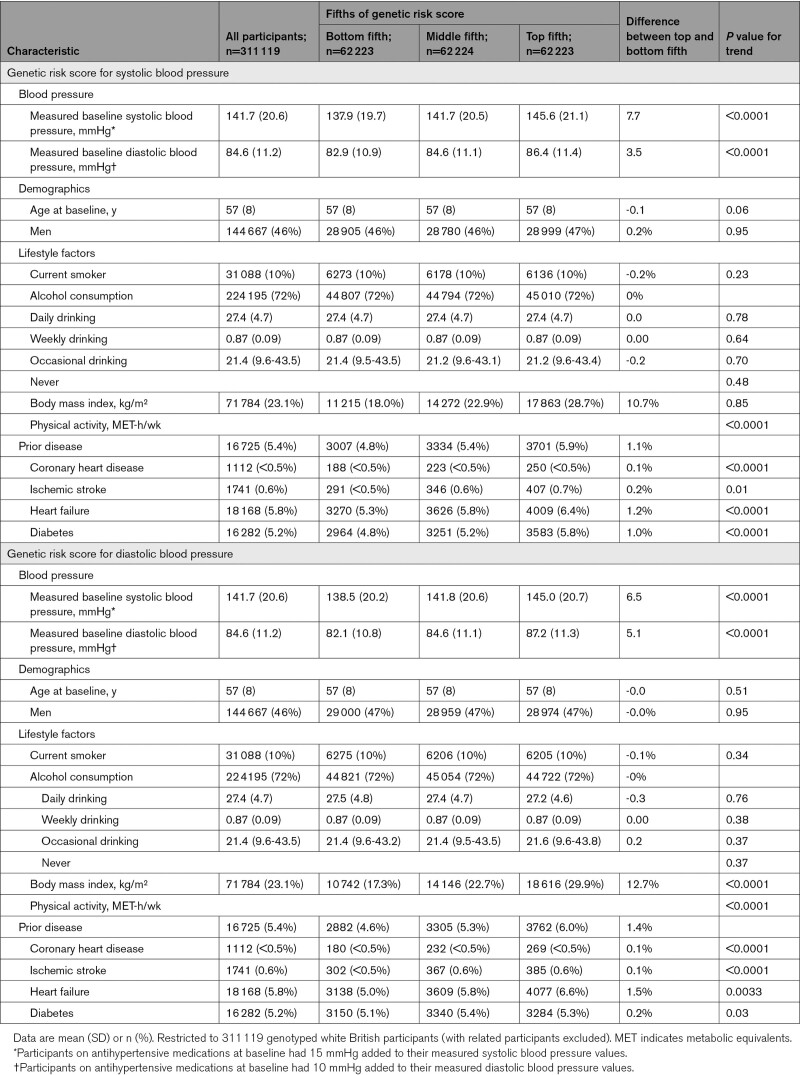
Baseline Characteristics of UK Biobank Participants, by Fifths of Genetic Risk Scores for Systolic and Diastolic Blood Pressure

### Associations of BP and Other Characteristics With GRS

For the systolic BP GRS, the difference in mean systolic and diastolic BP between top and bottom fifths of the GRS were 7.7 and 3.5 mmHg (equivalent to 0.37 and 0.31 SDs), respectively. For the diastolic BP GRS, the difference in mean systolic and diastolic BP between top and bottom fifth of the GRS was 6.5 and 5.1 mmHg (equivalent to 0.32 and 0.46 SDs), respectively (Table and Figure S2).

Age, sex, lifestyle factors, and measures of anthropometry were all similar across fifths of both GRSs. An expected higher prevalence of prior vascular disease with higher genetically predicted BP was observed: 4009 (6.4%) for the top fifth of the systolic BP GRS versus 3270 (5.3%) for the bottom fifth: difference 1.2%. There was also a higher prevalence of diabetes among those with higher genetically predicted BP, with a larger association for the systolic BP GRS than the diastolic BP GRS (differences between top and bottom fifths of the systolic and diastolic GRSs of 1.0% and 0.2%, respectively).

### Effect of Genetically Predicted Differences in BP on the Odds of CKD

**Figure 1.**
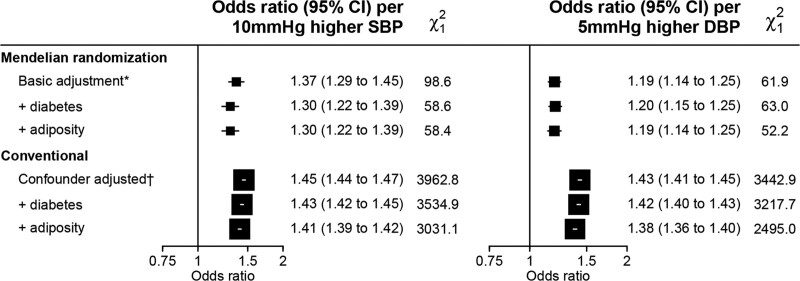
**Association of blood pressure with chronic kidney disease.** Chronic kidney disease defined as long−term kidney replacement therapy, estimated glomerular filtration rate <60 mL (min·1.73m^2^), or urinary albumin:creatinine ratio ≥3 mg/mmol. Analyses included 311 119 participants with 21 623 cases of chronic kidney disease. DBP indicates diastolic blood pressure; and SBP, systolic blood pressure. *Mendelian randomization analyses adjusted for age, age², sex, measured body mass index, top 18 principal components, and array. Multivariable Mendelian randomization analyses also adjusted for genetic effect of the blood pressure single nucleotide polymorphisms on diabetes, body mass index, and waist-to-hip ratio. †Conventional analyses adjusted for age, sex, ethnicity, education, region, deprivation index, smoking status, drinking status, and physical activity.

21 623 participants had evidence of CKD at recruitment: 7781 (2.5%) with reduced glomerular filtration and 15 500 (5.0%) with albuminuria (Table S3). Each genetically predicted 10 mmHg higher systolic BP and 5 mmHg higher diastolic BP was associated with a 37% (OR [odds ratio], 1.37 [95% CI, 1.29–1.45] and 19% [1.19; 1.14–1.25]) higher odds of CKD, respectively (Figure 1 and Figure S3).

**Figure 2.**
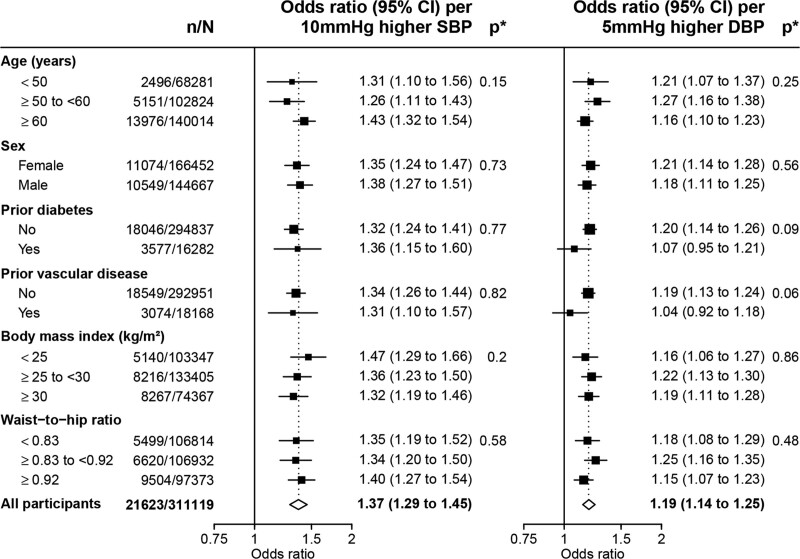
**Association of genetically predicted blood pressure with chronic kidney disease, by selected characteristics.** Chronic kidney disease defined as long-term kidney replacement therapy, estimated glomerular filtration rate <60 mL (mL/min/1.73m^2^), or urinary albumin:creatinine ratio ≥3 mg/mmol. Analyses adjusted for age, age², sex, measured body mass index, top 18 principal components, and array. DBP indicates diastolic blood pressure; and SBP, systolic blood pressure. *P value for test of heterogeneity or trend.

Sensitivity analyses showed that adjustment for the effects of the BP SNPs on type 2 diabetes, BMI, and WHR only modestly attenuated the odds ratio for genetically predicted 10 mmHg higher systolic BP to 1.30 (95% CI, 1.22–1.39). The odds ratio for genetically predicted 5 mmHg higher diastolic BP was essentially unchanged by such adjustments (Figure 1). Genetic BP-CKD associations were similar irrespective of age, sex, and the presence or absence of factors that predispose to dysregulated renal blood flow homeostasis, including history of diabetes, vascular disease, and level of adiposity (Figure 2). Sensitivity analyses stratifying by residual characteristics (where the genetic contribution to the characteristic has been removed) were not materially different (Figure S4).

After excluding 90 SNPs from systolic BP GRS and 67 SNPs from diastolic BP GRS that explain more variation in kidney function than BP, or in genes with differential expression in the kidney, or associated with the renin-angiotensin-aldosterone system or disordered kidney development/morphology/physiology, the association between BP and risk of CKD was, if anything, somewhat stronger (Figure S5). Results were also unaffected by removing 19 SNPs associated with diabetes (data not shown). Sensitivity analyses performed using 2-sample summary data approaches were consistent with the MR analyses presented in Figure 1 (Figure S6). For systolic BP, the intercept from the MR-Egger analyses suggested the potential presence of some directional pleiotropy (odds ratio, 1.004 [95% CI, 1.001–1.007]; *p* =0.02) albeit the bias-adjusted effect estimate remained significant, with each genetically predicted 10 mmHg higher systolic BP still associated with 20% higher odds of CKD (1.20 [1.05–1.37]).

### Effect of Genetically Predicted Differences in BP on the Odds of Different Levels of eGFR

**Figure 3.**
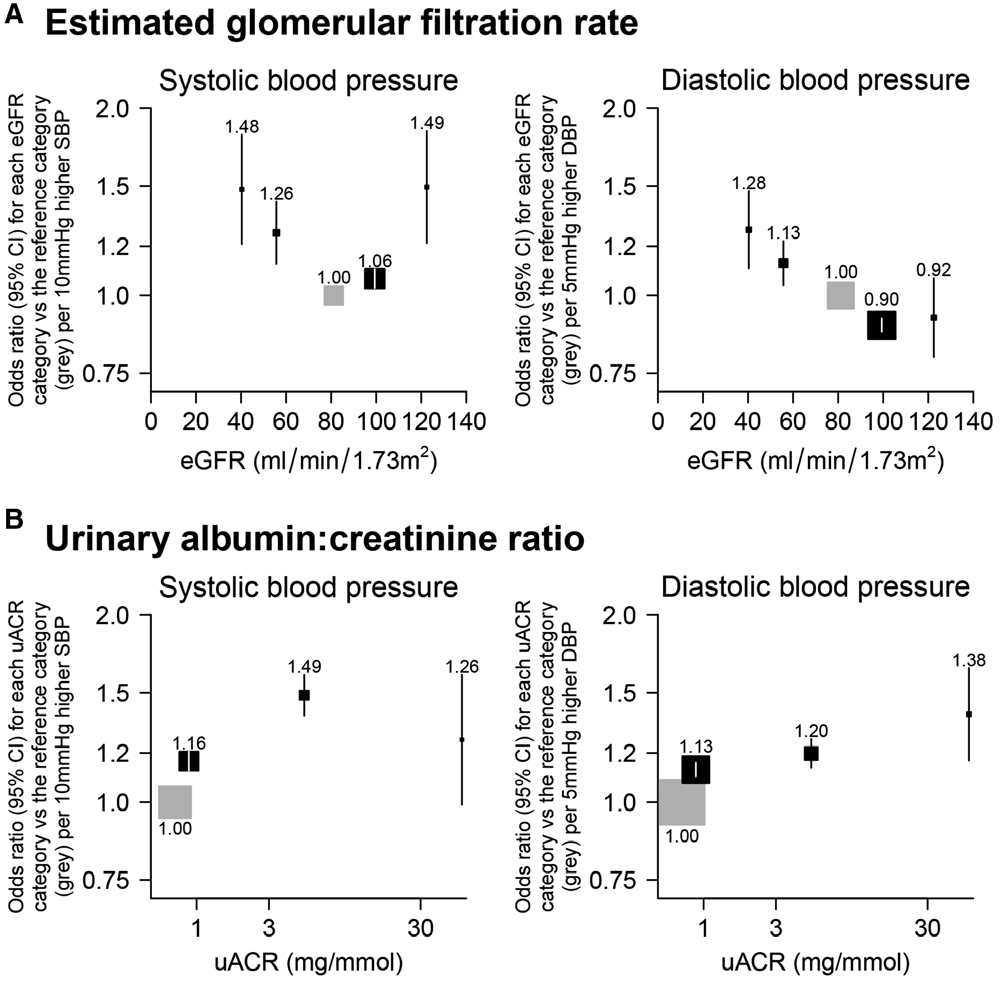
**Association of genetically predicted blood pressure with estimated glomerular filtration rate (eGFR) and urinary albumin:creatinine ratio (uACR) categories.** Odds ratios per 10 mmHg higher SBP and 5mmHg higher DBP for (**A**) eGFR and (**B**) uACR categories relative to the reference categories (indicated by the gray boxes) are shown. Analyses adjusted for age, age², sex, measured body mass index, top 18 principal components, and array.

Systolic BP-eGFR models revealed marked U-shaped associations. Higher systolic BP was associated both with the odds of decreased kidney function (ie, eGFR <60 mL [mL/min/1.73m^2^]) versus normal kidney function and with the odds of having an eGFR ≥90 mL (mL/min/1.73m^2^) versus normal kidney function (Figure 3). In particular, there were 1828 participants with direct evidence of hyperfiltration (ie, eGFR ≥120 mL [mL/min/1.73m^2^]); each 10 mmHg higher systolic BP was associated with a 49% higher odds of hyperfiltration versus normal kidney function (1.49 [1.21–1.84]: Figure 3). Somewhat in contrast, although each genetically predicted 5 mmHg higher diastolic BP was associated with higher odds of decreased kidney function, it was not associated with greater odds of hyperfiltration: odds ratio per 5 mmHg higher genetically predicted diastolic BP was 0.92 (0.80–1.07: Figure 3).

The positive systolic BP-hyperfiltration association was attenuated but still present after adjustment for the effects of the BP SNPs on type 2 diabetes, BMI, and WHR (1.37 [1.09–1.72]: Figure S7) and was at least as large among those without diabetes or without vascular disease (Figure S8). BP-hyperfiltration associations were absent in people with diabetes, but such analyses were based on only 129 participants with hyperfiltration.

After excluding 90 SNPs from systolic BP GRS and 67 SNPs from diastolic BP GRS that explain more variation in kidney function than BP, or in genes with differential expression in the kidney, or associated with renin-angiotensin-aldosterone system or disordered kidney development/morphology/physiology, the association between systolic BP and hyperfiltration was consistent (Figure S5) with the results in Figure 3. Results were also unaffected by removing 19 SNPs associated with diabetes (data not shown). Sensitivity analyses performed using 2-sample summary data approaches were also consistent with the results shown in Figure 3, and there was no evidence of directional pleiotropy when using the MR-Egger approach (Figure S6).

### Effect of Genetically Predicted BP on the Odds of Different Levels of Albuminuria

For the albuminuria-based outcomes, an exposure-response relationship was apparent with both higher genetically predicted systolic and diastolic BP associated with evidence of increasing odds of higher albuminuria categories (Figure 3). This relationship was unchanged by adjustment for the effects of the BP SNPs on type 2 diabetes, BMI, and WHR (Figure S7).

### Effect of Genetically Predicted Differences in BP on the Odds of AKI

10 122 (3.3%) participants had a record of hospitalization with AKI. Each genetically predicted 10 mmHg higher systolic BP was associated with a 15% (1.15 [1.04–1.28]) increased odds of AKI (Figure S9). Associations were similar irrespective of baseline eGFR category (trend test *p *=0.14), and the other subgroups (Figure S10). There was no association between genetically predicted diastolic BP and odds of AKI (1.01 [0.94–1.09]).

### Observational Associations of Measured BP With Kidney Outcomes

Conventional associations between measured BP with risk of CKD were also positive but were found to be somewhat stronger than genetic analyses (Figure 1). Conventional cross-sectional analyses of measured BP and levels of eGFR and uACR demonstrated similarly shaped exposure-response relationships to genetic analyses, although the OR for uACR were somewhat larger than MR analyses and appear to be driving the stronger association between measured BP and CKD (Figures S3 and S11).

## Discussion

We aimed to assess whether moderate life-long differences in BP are causally related to CKD by harnessing the scale of genetic information within UK Biobank and careful selection of kidney outcomes. We found evidence of a U-shaped association between genetically predicted higher systolic BP and eGFR. This strengthens the hypothesis that life-long higher systolic BP is a causal risk factor for incident CKD (including both lower eGFR and albuminuria), as well as glomerular hyperfiltration (which may be a precursor for kidney function decline). Each 10 mmHg higher genetically predicted systolic BP was associated with higher odds of CKD, and separately hyperfiltration, by about one-third. These associations seemed similar in size in people with or without conditions considered to disrupt renal blood flow autoregulation, including diabetes mellitus, obesity, or vascular disease. These results suggest physiological autoregulation may not afford complete protection against genetically predicted differences in BP.

Our results challenge the conclusions from the largest MR studies which reported no evidence of association between genetically predicted higher BP and differences in kidney function.^7,17^ The apparently discrepant findings may be due to glomerular hyperfiltration being a precursor to kidney function decline and more advanced stages of CKD, and the consequent nonlinear associations between BP and eGFR (which were not accounted for in previous MR experiments). Our finding of genetically predicted higher systolic BP being associated with glomerular hyperfiltration are consistent with the observed effects of intensive BP lowering in the SPRINT (Systolic BP Intervention Trial). In the SPRINT population of adults without diabetes, allocation to intensive BP lowering achieved an average systolic BP of 121 mmHg (compared with 136 mmHg in those on standard BP lowering), and an average 3 mL (mL/min/1.73m^2^) difference in eGFR. The eGFR decline/difference among those allocated intensive BP lowering was associated with reductions in albuminuria and filtered markers of tubular function, and no increase in markers of tubular injury, suggesting hemodynamic changes in the kidney, and perhaps a reversal of single nephron hyperfiltration.^8,45,46^

Our results challenge the notion that renal blood flow autoregulation fully protects against moderate elevations in systolic BP.^9^ The odds of CKD and hyperfiltration with life-long genetically predicted higher systolic BP were at least as large among those without diabetes, without preexisting vascular disease, and among those with ideal levels of adiposity. The lack of a detectable genetic association between diastolic BP and hyperfiltration raises the hypothesis that peak glomerular perfusion pressure rather than mean perfusion pressure may be key to glomerular barotrauma.

The present study benefits from UK Biobank’s large size and the use of methods that are less susceptible to residual confounding and reverse causality, but some limitations may exist. First, it is possible that some of the BP GRS included SNPs exert a direct effect on the kidney or its vasculature independent of their effect on BP. However, we carefully sought and excluded SNPs that were in, or close to, genes differentially expressed in the kidney, and any SNPs reported as being involved in TGF-beta signaling,^34^ the renin-angiotensin system, or disordered kidney development, morphology, or physiology.^18^ Findings were unaltered after exclusion of these SNPs from analyses. Second, analyses were based on single measurements of eGFR and albuminuria, meaning BP-CKD associations may be underestimated. Third, although sensitivity analyses stratified by residual characteristics found no clear evidence of such a collider bias, stratification for subgroup analyses could conceivably lead to its introduction and need cautious interpretation. Lastly, the study was restricted to White British adults, meaning results may not be generalizable to other populations.

In conclusion, the use of nonlinear MR models shows that life-long elevation of BP is a cause of both decreased kidney function which is characteristic of progressive CKD, and glomerular hyperfiltration which may be a precursor for kidney function decline. These results contrast previous findings from MR studies that erroneously assumed linear associations. Higher genetically predicted systolic BP was more strongly related to CKD risk and hyperfiltration than higher diastolic BP, with risks evident in the presence or absence of diabetes, obesity, and vascular disease which predispose to CKD or hyperfiltration. These analyses suggest early active management of moderate systolic hypertension could reduce long-term risk of CKD, even in people without diabetes, obesity, or established cardiovascular disease.

## Perspectives

The presented analyses from UK Biobank data suggest a causal role for life-long small increases in BP in the development of CKD and glomerular hyperfiltration (a precursor to CKD). Genetically predicted BP associates with CKD outcomes based on decreased kidney function, abnormally increased kidney function, and/or albuminuria. These findings raise a convincing hypothesis that physiological autoregulation may not afford complete renal protection against moderate BP elevations. Consequently, considering early active management of moderate systolic hypertension in all adults—and not just individuals with diabetes, obesity, or other CKD risk factors—may be an important population health strategy. Replicating these findings in other cohorts, including cohorts with a larger number of relevant advanced CKD cases and in non-White populations are important research priorities for renal epidemiology.

## Article Information

### Acknowledgments

This research has been conducted using the open access UK Biobank Resource (application number 15595), with data available from: https://www.ukbiobank.ac.uk/. This article has not been published previously in whole or part.

### Author Contributions

N. Staplin, W.G. Herrington, R. Haynes, J.C. Hopewell conceived the study and developed its design; NS performed statistical analyses; F. Murgia and M. Ibrahim performed bioinformatics analyses and functional mapping; N. Staplin and J.C. Hopewell had full access to the data; N. Staplin, W.G. Herrington wrote the first draft of the article; and all authors contributed to data interpretation and revision of the article.

### Sources of Funding

Clinical Trial Service Unit and Epidemiological Studies Unit (CTSU) has a staff policy of not accepting honoraria or other payments from the pharmaceutical industry, except for the reimbursement of costs to participate in scientific meetings. The Medical Research Council Population Health Research Unit at the University of Oxford, which is part of the CTSU, receives core funding from the United Kingdom Medical Research Council. We also acknowledge support from Health Data Research UK, National Institute for Health and Care Research Oxford Biomedical Research Centre, and British Heart Foundation Centre for Research Excellence, Oxford. W.G. Herrington and K.R. Bull are both supported by Medical Research Council Kidney Research UK Professor David Kerr Clinician Scientist Awards. J.C. Hopewell is supported by a personal fellowship from the British Heart Foundation (FS/14/55/30806).

### Disclosures

For the purpose of open access, the author(s) has applied a Creative Commons Attribution (CC BY) licence to any Author Accepted Manuscript version arising.

## Supplementary Material


