# Supplementary material for: Determining the Relationship between Blood Pressure, Kidney Function, and Chronic Kidney Disease: Insights from Genetic Epidemiology
Source: Hypertension. Author manuscript; Available in PMC 2022 Dec 1. (PMC9640248; doi:10.1161/HYPERTENSIONAHA.122.19354)
Supplement: Supplementary Material [file EMS152991-supplement-Supplementary_Material.pdf]

ONLINE SUPPLEMENT

Determining the Relationship between Blood Pressure, Kidney Function, and Chronic Kidney Disease: Insights from Genetic Epidemiology

Natalie Staplin PhD*[1,2,3], William G. Herrington MD*[1,2,4,5], Federico Murgia PhD[2,3], Maysson Ibrahim PhD[2,3], Katherine R. Bull DPhil[4,6], Parminder Judge PhD[2,4], Sarah Y.A. Ng MBChB[1,2] Michael Turner MBChB[1,2,4], Doreen Zhu MBChB[1,2,4], Jonathan Emberson PhD[1,2,3], Martin J. Landray FMedSci[1,2,3,5,7], Colin Baigent FMedSci[1,2], Richard Haynes DM[†1,2,4], Jemma C. Hopewell PhD[†2,3].

*/† joint contribution

[1] Medical Research Council Population Health Research Unit at the University of Oxford, Nuffield Department of Population Health (NDPH), Oxford, UK

[2] Clinical Trial Service Unit and Epidemiological Studies Unit, NDPH, University of Oxford, Oxford, UK

[3] Big Data Institute, Li Ka Shing Centre for Health Information and Discovery, University of Oxford, Oxford, UK

[4] Oxford Kidney Unit, Churchill Hospital, Oxford, UK

[5] Health Data Research UK, University of Oxford, Oxford, UK

[6] Nuffield Department of Medicine, University of Oxford, Oxford, UK

[7] National Institute for Health Research Oxford Biomedical Research Centre, University of Oxford, Oxford, UK

Correspondence to: Associate Professor Natalie Staplin or Professor Jemma Hopewell

MRC Population Health Research Unit at the University of Oxford,

Big Data Institute, Old Road Campus,

Roosevelt Drive, Oxford OX3 7LF, UK

Email: natalie.staplin@ndph.ox.ac.uk or jemma.hopewell@ndph.ox.ac.uk

Phone: +44 1865 743743; Fax: +44 1865 743985

SUPPLEMENTAL TEXT

Supplemental methods

Functional annotations and tissue specificity enrichment analysis

The SNPs were annotated using the Whole Genome Sequencing Annotation (WGSA) pipeline according to the GRCh37 reference genome.(1) WGSA annotates variants with respect to their functional consequences on genes and add external biological knowledge, such as functional prediction scores and regulatory region-centric elements from different epigenomic projects. To simplify the consequence interpretation, the most damaging consequence based on Ensembl variant effect predictor (VEP) tool for each variant was used.(2) Using the list of functionally annotated genes, we performed a tissue specificity enrichment analysis using the GENE2FUNC function as implemented in the Functional Mapping and Annotation pipeline (FUMA).(3) Briefly, FUMA pre-computes a list of differentially expressed genes (DEGs) in 53 tissue types using GTEx V7 data, a tissue-based RNA repository based on post-mortem samples. DEG sets were pre-calculated by performing a two-sided t-test per gene per tissue against all other tissues. Genes with P-value ≤ 0.05 after Bonferroni correction and an absolute log fold change ≥0.58 were defined as differentially expressed in a given tissue compared to others. Input genes were then tested for enrichment against each of the pre-defined DEGs sets using the hypergeometric test where background genes are those with an average expression value >1 in at least one of the tissues (Figure S1).

Supplemental references

1. Liu X, White S, Peng B, Johnson AD, Brody JA, Li AH, et al. WGSA: an annotation pipeline for human genome sequencing studies. J Med Genet. 2016;53(2):111-2.
2. McLaren W, Gil L, Hunt SE, Riat HS, Ritchie GR, Thormann A, et al. The Ensembl Variant Effect Predictor. Genome Biol. 2016;17(1):122.
3. Watanabe K, Taskesen E, van Bochoven A, Posthuma D. Functional mapping and annotation of genetic associations with FUMA. Nat Commun. 2017;8(1):1826.

Table S1: SNPs included in genetic risk scores for systolic and diastolic blood pressure

SNP	Chromosome: Position	Effect allele	Weight	Functional Gene	Differential expression in kidney	Disordered kidney morphology/ physiology	TGF-beta signalling	Renin-angiotensin system	Steiger filtering	Excluded in sensitivity analyses
**Genetic risk score for systolic blood pressure**										
rs10008637	4:77414144	T	0.1355	SHROOM3	NO	NO	NO	NO	YES	YES
rs10048404	18:54578482	T	-0.1412	WDR7	YES	NO	NO	NO	NO	YES
rs10059921	5:87514515	T	-0.3732	TMEM161B	NO	NO	NO	NO	NO	NO
rs10069690	5:1279790	T	0.2151	TERT	NO	NO	NO	NO	NO	NO
rs10077885	5:114390121	A	-0.2465	TRIM36	NO	NO	NO	NO	NO	NO
rs10224002	7:151415041	A	-0.2375	PRKAG2	NO	NO	NO	NO	YES	YES
rs10233127	7:30933453	A	0.3906	AQP1	NO	NO	NO	NO	NO	NO
rs10274928	7:28142088	A	0.1505	JAZF1	YES	NO	NO	NO	NO	YES
rs1036902	17:58950791	T	-0.1786	BCAS3	NO	NO	NO	NO	YES	YES
rs10409243	19:10332988	T	-0.1088	CTD-2369P2.2	YES	NO	NO	NO	YES	YES
rs1043069	1:180859368	T	0.1287	XPR1	NO	NO	NO	NO	NO	NO
rs10437954	12:58003922	A	-0.2398	ARHGEF25	NO	NO	NO	NO	NO	NO
rs1044822	2:230629138	T	-0.1852	TRIP12	YES	NO	NO	NO	NO	YES
rs10743086	11:8774923	A	-0.14	ST5	NO	NO	NO	NO	NO	NO
rs1076485	11:116772441	T	0.2665	SIK3	YES	NO	NO	NO	NO	YES
rs10778174	12:102838996	A	-0.2289	IGF1	YES	NO	NO	NO	NO	YES
rs10782230	6:126228512	A	0.1776	NCOA7	NO	NO	NO	NO	NO	NO
rs10858966	12:90567026	C	0.248	LINC00936	YES	NO	NO	NO	NO	YES
rs10922502	1:89360158	A	-0.2283	GTF2B	YES	NO	NO	NO	NO	YES
rs11031051	11:30355707	A	-0.127	ARL14EP	YES	NO	NO	NO	NO	YES
rs11112548	12:105871914	A	0.3673	C12orf75	YES	NO	NO	NO	NO	YES
rs111245230	9:113169775	T	-0.6917	SVEP1	NO	NO	NO	NO	NO	NO
rs11128722	3:14958126	A	-0.2518	FGD5	NO	NO	NO	NO	NO	NO
rs11159091	14:75074316	A	0.1433	LTBP2	YES	NO	NO	NO	NO	YES
rs11191548	10:104846178	T	1.0233	CNNM2	NO	NO	NO	NO	YES	YES
rs11197813	10:118523933	A	-0.1765	HSPA12A	NO	NO	NO	NO	NO	NO
rs112184198	10:102604514	A	-0.5331	PAX2	YES	YES	NO	NO	NO	YES
rs112280096	17:79367409	A	-0.1169	RP11-1055B8.4	NO	NO	NO	NO	NO	NO
rs11229457	11:58207203	T	-0.2886	OR5B12	NO	NO	NO	NO	NO	NO
rs1126930	12:49399132	C	0.5757	PRKAG1	NO	NO	NO	NO	NO	NO
rs1133400	10:134459388	A	-0.193	INPP5A	YES	NO	NO	NO	NO	YES
rs11537751	11:47587452	T	0.3936	NDUFS3	NO	NO	NO	NO	NO	NO
rs11571376	12:1059556	C	-0.2044	RAD52	NO	NO	NO	NO	NO	NO
rs11623535	14:72462381	A	0.1885	RGS6	NO	NO	NO	NO	NO	NO
rs11629850	15:40317075	A	0.1417	EIF2AK4	YES	NO	NO	NO	NO	YES
rs11632436	15:86295286	C	0.1738	RP11-158M2.4	NO	NO	NO	NO	NO	NO
rs11634028	15:76276150	A	0.3107	NRG4	YES	NO	NO	NO	YES	YES
rs11701033	21:33788341	C	-0.2465	EVA1C	YES	NO	NO	NO	NO	YES
rs1173771	5:32815028	A	-0.5227	NPR3	YES	NO	NO	NO	NO	YES
rs11771693	7:150050111	A	0.1368	RARRES2	NO	NO	NO	NO	NO	NO
rs11876341	18:48799991	A	-0.2406	MEX3C	YES	NO	NO	NO	NO	YES
rs11953630	5:157845402	T	-0.4463	Mir_186	NO	NO	NO	NO	NO	NO
rs12042924	1:197297417	T	-0.1235	CRB1	NO	NO	NO	NO	NO	NO
rs12454712	18:60845884	T	0.2258	BCL2	NO	NO	NO	NO	NO	NO
rs12473688	2:185033470	A	0.1425	ZNF804A	NO	NO	NO	NO	NO	NO

rs12511987	4:46595623	T	-0.1817	COX7B2	NO	NO	NO	NO	NO	NO
rs12572586	10:74751579	T	-0.3177	P4HA1	NO	NO	NO	NO	NO	NO
rs12638085	3:30405936	A	0.2004	TGFBR2	NO	NO	NO	NO	NO	NO
rs12694277	2:213188795	T	-0.1284	ERBB4	YES	NO	NO	NO	NO	YES
rs12703989	7:140238048	A	0.1501	DENND2A	NO	NO	NO	NO	NO	NO
rs1275988	2:26914364	T	-0.5157	KCNK3	NO	NO	NO	NO	NO	NO
rs12946454	17:43208121	A	-0.3193	PLCD3	YES	NO	NO	NO	NO	YES
rs12958173	18:42141977	A	0.3518	BC051727	NO	NO	NO	NO	NO	NO
rs13112725	4:106911742	C	0.397	NPNT	YES	NO	NO	NO	NO	YES
rs13149209	4:89750668	T	0.196	FAM13A	NO	NO	NO	NO	NO	NO
rs13179413	5:55868097	T	0.1656	AC022431.2	YES	NO	NO	NO	NO	YES
rs13238550	7:131059056	A	0.1695	MKLN1	YES	NO	NO	NO	NO	YES
rs1332813	9:9350706	T	0.1453	PTPRD	NO	NO	NO	NO	NO	NO
rs13359291	5:122476457	A	0.4005	PRDM6	NO	NO	NO	NO	NO	NO
rs13420463	2:37517566	A	0.2751	PRKD3	YES	NO	NO	NO	NO	YES
rs1344653	2:19730845	A	-0.1568	OSR1	YES	NO	NO	NO	NO	YES
rs1347345	4:95938386	A	-0.1765	BMPR1B	NO	NO	NO	NO	NO	NO
rs1375564	3:85656311	T	0.1884	CADM2	NO	NO	NO	NO	NO	NO
rs138877676	19:50935809	T	-0.6453	MYBPC2	NO	NO	NO	NO	NO	NO
rs139354822	2:242344695	T	0.442	FARP2	NO	NO	NO	NO	NO	NO
rs143112823	3:154707967	A	-0.4019	MME	YES	NO	NO	NO	NO	YES
rs1446468	2:164963486	T	-0.487	AC092684.1	NO	NO	NO	NO	NO	NO
rs1551355	17:30032420	T	0.1618	RP11-805L22.1	NO	NO	NO	NO	NO	NO
rs1563788	6:43308363	T	0.3062	ZNF318	YES	NO	NO	NO	NO	YES
rs1585453	11:46884713	A	-0.4052	LRP4-AS1	NO	NO	NO	NO	NO	NO
rs1694068	5:53283630	A	0.1739	ARL15	NO	NO	NO	NO	NO	NO
rs17035181	4:157678511	T	0.271	PDGFC	NO	NO	NO	NO	NO	NO
rs17115145	14:30122409	T	0.1159	CTD-2503I6.1	NO	NO	NO	NO	NO	NO
rs17249754	12:90060586	A	-0.8015	ATP2B1	YES	NO	NO	NO	NO	YES
rs17367504	1:11862778	A	0.7774	MTHFR	YES	NO	NO	NO	NO	YES
rs17396055	1:94730954	A	-0.1654	ARHGAP29	NO	NO	NO	NO	NO	NO
rs17477177	7:106411858	T	-0.5642	RP5-884M6.1	NO	NO	NO	NO	NO	NO
rs17638167	19:11584818	T	-0.5228	CTC-398G3.6	NO	NO	NO	NO	NO	NO
rs184457	9:131940019	A	-0.1527	IER5L	NO	NO	NO	NO	NO	NO
rs1882289	3:114461208	A	-0.2289	ZBTB20	NO	NO	NO	NO	NO	NO
rs1882961	21:16556367	T	0.2147	NRIP1	NO	NO	NO	NO	NO	NO
rs1891730	9:130309028	T	-0.1733	FAM129B	NO	NO	NO	NO	NO	NO
rs189267552	3:20073193	A	-0.5985	KAT2B	YES	NO	NO	NO	NO	YES
rs190194639	11:34068037	T	0.2211	CAPRIN1	NO	NO	NO	NO	NO	NO
rs1906672	8:38130025	A	0.2227	WHSC1L1	YES	NO	NO	NO	NO	YES
rs1986971	8:10268736	A	0.1991	MSRA	YES	NO	NO	NO	NO	YES
rs2014912	4:86715670	T	0.5122	ARHGAP24	YES	YES	NO	NO	NO	YES
rs2024385	12:12888438	A	-0.1588	APOLD1	YES	NO	NO	NO	NO	YES
rs2240736	17:59485393	T	0.4265	TBX2	YES	NO	NO	NO	YES	YES
rs2291435	4:38387395	T	-0.2419	RP11-83C7.1	NO	NO	NO	NO	NO	NO
rs2300481	2:66782467	T	0.1887	MEIS1	NO	NO	NO	NO	NO	NO
rs231708	4:2694773	C	-0.1252	FAM193A	YES	NO	NO	NO	NO	YES
rs246973	5:68007803	T	0.1721	SLC30A5	NO	NO	NO	NO	NO	NO
rs2498586	6:118026126	T	-0.1617	NUS1	NO	NO	NO	NO	NO	NO
rs260508	1:2187085	T	0.1297	SKI	YES	NO	NO	NO	NO	YES

rs2610990	4:18008232	A	-0.1915	LCORL	NO	NO	NO	NO	NO	NO
rs2613765	19:5066330	A	-0.2304	KDM4B	YES	NO	NO	NO	NO	YES
rs2688716	11:54835623	T	-0.2062	TRIM48	NO	NO	NO	NO	NO	NO
rs2745599	6:1613686	A	0.1464	FOXC1	YES	NO	NO	NO	NO	YES
rs2759308	15:81016227	A	0.2592	ABHD17C	NO	NO	NO	NO	NO	NO
rs2807337	1:22577371	T	0.1551	WNT4	NO	NO	NO	NO	NO	NO
rs28377357	2:112769721	A	-0.1335	MERTK	NO	NO	NO	NO	NO	NO
rs28558491	2:187816321	T	-0.1419	ZSWIM2	NO	NO	NO	NO	NO	NO
rs28558845	9:4334791	C	-0.1476	GLIS3	YES	NO	NO	NO	NO	YES
rs28578714	22:50727921	T	0.1761	PLXNB2	NO	NO	NO	NO	NO	NO
rs2898290	8:11433909	T	0.3419	LINC00208	NO	NO	NO	NO	NO	NO
rs2920899	2:55279681	T	0.1579	RTN4	NO	NO	NO	NO	NO	NO
rs2972146	2:227100698	T	0.2486	LOC646736	NO	NO	NO	NO	NO	NO
rs3121685	5:65662133	T	-0.1302	SREK1	NO	NO	NO	NO	NO	NO
rs33996239	1:203109801	T	-0.3263	ADORA1	NO	NO	NO	NO	NO	NO
rs34072724	7:130432469	A	-0.1735	KLF14	NO	NO	NO	NO	NO	NO
rs34130368	10:48411796	T	-0.2205	GDF2	NO	NO	NO	NO	NO	NO
rs34413141	18:777282	A	-0.2124	YES1	YES	NO	NO	NO	NO	YES
rs34430710	17:56876627	A	-0.1972	PPM1E	YES	NO	NO	NO	NO	YES
rs34756251	15:100192540	T	-0.2265	MEF2A	YES	NO	NO	NO	NO	YES
rs347591	3:11290122	T	0.2842	HRH1	YES	NO	NO	NO	NO	YES
rs35410524	6:96885405	T	0.2999	UFL1-AS1	NO	NO	NO	NO	NO	NO
rs35450617	16:6889675	T	-0.1298	RBFOX1	NO	NO	NO	NO	NO	NO
rs35590893	2:43716933	A	-0.1706	THADA	NO	NO	NO	NO	NO	NO
rs35783704	8:105966258	A	-0.5219	RP11-127H5.1	NO	NO	NO	NO	YES	YES
rs3741378	11:65408937	T	-0.4169	SIPA1	NO	NO	NO	NO	NO	NO
rs3743157	15:85680532	A	0.1997	PDE8A	NO	NO	NO	NO	NO	NO
rs3820068	1:15798197	A	0.3361	CELA2B	NO	NO	NO	NO	YES	YES
rs4129585	8:143312933	A	0.1541	TSNARE1	NO	NO	NO	NO	NO	NO
rs4247374	19:7252756	T	-0.5063	INSR	NO	NO	NO	NO	NO	NO
rs4480845	17:1958609	T	0.1473	HIC1	NO	NO	NO	NO	NO	NO
rs4499560	3:70920485	A	-0.1637	FOXP1	YES	NO	NO	NO	NO	YES
rs4598218	8:129483956	T	0.1578	RP11-89M16.1	NO	NO	NO	NO	NO	NO
rs4651224	1:184585182	T	0.186	C1orf21	YES	NO	NO	NO	NO	YES
rs4728142	7:128573967	A	-0.2155	IRF5	YES	NO	NO	NO	NO	YES
rs4754196	11:107096777	A	-0.1756	CWF19L2	NO	NO	NO	NO	NO	NO
rs4873492	8:51947549	T	0.2131	SNTG1	NO	NO	NO	NO	NO	NO
rs4925159	17:18185510	A	0.2482	TOP3A	YES	NO	NO	NO	NO	YES
rs4926499	1:249155909	C	0.2925	AL672294.1	NO	NO	NO	NO	NO	NO
rs4980515	11:63744609	T	0.1722	AP000721.4	NO	NO	NO	NO	NO	NO
rs5219	11:17409572	T	0.32	KCNJ11	YES	NO	NO	NO	NO	YES
rs55701159	2:25139596	T	0.2999	ADCY3	YES	NO	NO	NO	NO	YES
rs55780018	2:208526140	T	-0.3278	AC079767.4	NO	NO	NO	NO	NO	NO
rs56352451	10:5804865	T	0.1563	FAM208B	YES	NO	NO	NO	NO	YES
rs590198	3:135953729	A	0.1155	PCCB	NO	NO	NO	NO	NO	NO
rs6021247	20:50108980	A	0.1506	NFATC2	YES	NO	NO	NO	NO	YES
rs6031435	20:42797358	A	-0.2268	JPH2	YES	NO	NO	NO	NO	YES
rs606950	13:22298923	A	0.1585	FGF9	YES	NO	NO	NO	NO	YES
rs62373688	5:127352807	A	0.2301	CTC-228N24.3	YES	NO	NO	NO	NO	YES
rs62491354	8:9730663	A	0.1582	TNKS	YES	NO	NO	NO	NO	YES

rs62523863	8:126520544	A	0.1643	RP11-136O12.2	NO	NO	NO	NO	NO	NO
rs6438857	3:124557643	T	0.0977	ITGB5	NO	NO	NO	NO	NO	NO
rs6504213	17:62381714	T	-0.1381	RPL31P57	NO	NO	NO	NO	NO	NO
rs6545155	2:50429861	T	0.1442	NRXN1	NO	NO	NO	NO	NO	NO
rs6557876	8:25900675	T	-0.3667	EBF2	NO	NO	NO	NO	NO	NO
rs6593297	7:56122058	A	0.1398	CCT6A	YES	NO	NO	NO	NO	YES
rs6595838	5:127868199	A	0.2361	FBN2	NO	NO	NO	NO	NO	NO
rs661348	11:1905292	T	-0.3417	LSP1	YES	NO	NO	NO	NO	YES
rs6723509	2:122000745	T	0.2018	TFCP2L1	YES	NO	NO	NO	NO	YES
rs67720684	2:18975439	A	0.2242	NT5C1B-RDH14	NO	NO	NO	NO	NO	NO
rs6788984	3:41107173	A	0.168	CTNNB1	NO	NO	NO	NO	NO	NO
rs67976715	11:68023742	C	0.1742	C11orf24	YES	NO	NO	NO	NO	YES
rs6911827	6:22130601	T	0.152	CASC15	NO	NO	NO	NO	NO	NO
rs6959688	7:1966831	A	-0.1323	MAD1L1	YES	NO	NO	NO	NO	YES
rs6963105	7:75097488	A	-0.1548	POM121C	NO	NO	NO	NO	NO	NO
rs6969780	7:27159136	C	0.3697	HOXA3	YES	NO	NO	NO	NO	YES
rs6996733	8:60535824	T	0.1484	SNORA51	NO	NO	NO	NO	NO	NO
rs7023828	9:128498594	T	-0.2125	PBX3	YES	NO	NO	NO	YES	YES
rs702395	5:140086677	T	0.115	VTRNA1-1	NO	NO	NO	NO	NO	NO
rs7045409	9:95201540	A	-0.1746	CENPP	NO	NO	NO	NO	NO	NO
rs7096563	10:133770229	A	0.1648	PPP2R2D	NO	NO	NO	NO	NO	NO
rs709668	5:96174186	A	-0.1743	CTD-2260A17.2	YES	NO	NO	NO	NO	YES
rs7129220	11:10350538	A	0.3919	AMPD3	NO	NO	NO	NO	NO	NO
rs7187540	16:85318302	A	-0.1431	LINC00311	NO	NO	NO	NO	NO	NO
rs72683923	14:50735947	T	1.0239	L2HGDH	NO	NO	NO	NO	NO	NO
rs72688070	8:81393697	T	-0.187	ZBTB10	NO	NO	NO	NO	NO	NO
rs72719160	4:144051276	A	-0.2509	RP11-284M14.1	NO	NO	NO	NO	NO	NO
rs72816333	2:60096560	A	0.2016	RP11-444A22.1	NO	NO	NO	NO	NO	NO
rs72844590	2:138421227	T	0.1893	THSD7B	NO	YES	NO	NO	NO	YES
rs72847885	2:86326717	A	0.1717	POLR1A	YES	NO	NO	NO	NO	YES
rs7297416	12:54443090	A	0.2816	HOXC4	YES	NO	NO	NO	NO	YES
rs73082337	3:49009570	C	0.1808	ARIH2	YES	NO	NO	NO	NO	YES
rs73099903	12:53440779	T	0.4218	TENC1	NO	NO	NO	NO	NO	NO
rs73187288	13:42738672	A	-0.2105	DGKH	NO	NO	NO	NO	NO	NO
rs7331680	13:115000650	T	0.3414	CDC16	YES	NO	NO	NO	NO	YES
rs7406910	17:46688256	T	-0.4877	HOXB7	YES	NO	NO	NO	NO	YES
rs7439567	4:138464842	T	0.1998	RP11-714L20.1	NO	NO	NO	NO	YES	YES
rs74774746	5:33411769	C	-0.1321	TARS	YES	NO	NO	NO	NO	YES
rs7514579	1:94051350	A	0.1443	BCAR3	NO	NO	NO	NO	NO	NO
rs7515635	1:42408070	T	0.2382	HIVEP3	YES	NO	NO	NO	NO	YES
rs7555285	1:209970355	C	0.1334	IRF6	NO	NO	NO	NO	NO	NO
rs7562	2:28635740	T	0.1555	FOSL2	NO	NO	NO	NO	NO	NO
rs75905900	11:55113534	A	0.328	OR4A16	NO	NO	NO	NO	NO	NO
rs76719272	1:156129796	T	-0.1708	SEMA4A	NO	NO	NO	NO	NO	NO
rs77413490	10:89681688	T	0.522	PTEN	YES	NO	NO	NO	NO	YES
rs7763294	6:140383733	T	-0.1418	RP3-332B22.1	NO	NO	NO	NO	NO	NO
rs78151625	3:158316726	T	-0.1451	MLF1	NO	NO	NO	NO	NO	NO
rs78474310	13:73826901	A	-0.5088	KLF5	NO	NO	NO	NO	NO	NO
rs78648104	6:50683009	T	-0.3571	TFAP2D	NO	NO	NO	NO	NO	NO
rs78998485	12:434755	C	-0.1262	KDM5A	YES	NO	NO	NO	YES	YES

rs7927515	11:76125330	A	0.1705	RP11-111M22.2	NO	NO	NO	NO	NO	NO
rs79523138	2:161368213	A	-0.301	RBMS1	NO	NO	NO	NO	NO	NO
rs79598313	1:27284913	T	0.456	C1orf172	YES	NO	NO	NO	NO	YES
rs7963801	12:79685226	T	-0.1327	SYT1	NO	NO	NO	NO	YES	YES
rs7976167	12:24210599	T	0.1337	SOX5	NO	NO	NO	NO	NO	NO
rs7988232	13:79808655	A	0.1392	RBM26	YES	NO	NO	NO	NO	YES
rs8014182	14:103859962	T	-0.3509	MARK3	NO	NO	NO	NO	NO	NO
rs8105753	19:31927547	A	0.1895	TSHZ3	YES	NO	NO	NO	NO	YES
rs839755	1:43856410	A	-0.1499	SZT2	NO	NO	NO	NO	NO	NO
rs848445	7:77572461	T	-0.196	PHTF2	YES	NO	NO	NO	YES	YES
rs871004	11:28512458	A	0.1329	RP11-22P4.1	NO	NO	NO	NO	NO	NO
rs880315	1:10796866	T	-0.5218	CASZ1	NO	NO	NO	NO	NO	NO
rs912434	13:47189928	T	0.1772	LRCH1	NO	NO	NO	NO	NO	NO
rs9302885	17:76799898	A	0.1367	USP36	NO	NO	NO	NO	NO	NO
rs932764	10:95895940	A	-0.3654	PLCE1	NO	YES	NO	NO	NO	YES
rs9368222	6:20686996	A	0.2337	CDKAL1	NO	NO	NO	NO	NO	NO
rs9401090	6:119113317	T	0.1762	MCM9	NO	NO	NO	NO	NO	NO
rs9526707	13:51489186	A	-0.1739	RNASEH2B	YES	NO	NO	NO	NO	YES
rs9532243	13:32191408	A	0.3595	RXFP2	NO	NO	NO	NO	NO	NO
rs9678851	2:27887034	A	-0.1135	SLC4A1AP	YES	NO	NO	NO	NO	YES
rs9857362	3:74710462	A	0.1104	CNTN3	NO	NO	NO	NO	NO	NO
rs9875380	3:132780356	T	-0.1456	TMEM108	NO	NO	NO	NO	NO	NO
rs9885632	6:131311909	T	0.1582	EPB41L2	YES	NO	NO	NO	NO	YES
rs9888615	14:53377540	T	-0.2356	FERMT2	NO	NO	NO	NO	NO	NO
**Genetic risk score for diastolic blood pressure**										
rs10062049	5:61553881	T	0.1821	KIF2A	YES	NO	NO	NO	NO	YES
rs10078021	5:75038431	T	-0.1218	POC5	YES	NO	NO	NO	NO	YES
rs10087782	8:141858620	T	0.0872	PTK2	NO	NO	NO	NO	NO	NO
rs1047030	8:22428708	A	0.1383	SORBS3	NO	NO	NO	NO	NO	NO
rs1060105	12:123806219	T	-0.1644	SBNO1	YES	NO	NO	NO	NO	YES
rs1063281	2:218668732	T	-0.1378	TNS1	NO	NO	NO	NO	NO	NO
rs1077795	19:17222584	A	0.0961	MYO9B	YES	NO	NO	YES	NO	YES
rs10850411	12:115387796	T	0.1856	TBX3	NO	NO	NO	NO	NO	NO
rs10906391	10:13523937	T	0.0976	BEND7	NO	NO	NO	NO	NO	NO
rs10943605	6:79655477	A	0.1552	IRAK1BP1	NO	NO	NO	NO	NO	NO
rs1098708	12:27321112	A	-0.0855	STK38L	YES	NO	NO	NO	NO	YES
rs11021221	11:95308854	A	-0.1445	5S_rRNA	NO	NO	NO	NO	NO	NO
rs11026586	11:22515533	A	0.1672	RP11-34N19.1	NO	NO	NO	NO	NO	NO
rs11030119	11:27728102	A	-0.1332	RP11-587D21.4	NO	NO	NO	NO	NO	NO
rs110419	11:8252853	A	0.1167	LMO1	NO	NO	NO	NO	NO	NO
rs11067763	12:116198341	A	0.1429	RP11-110L15.1	NO	NO	NO	NO	NO	NO
rs11080134	17:29161503	A	-0.1031	ATAD5	NO	NO	NO	NO	NO	NO
rs11168245	12:48204499	C	0.2022	HDAC7	NO	NO	NO	NO	NO	NO
rs1126464	16:89704365	C	0.2219	DPEP1	YES	NO	NO	NO	NO	YES
rs113134141	3:46861939	A	-0.1451	PRSS42	NO	NO	NO	NO	NO	NO
rs115245297	6:34244132	T	-0.3259	RP11-513I15.6	NO	NO	NO	NO	NO	NO
rs11556924	7:129663496	T	-0.142	ZC3HC1	NO	NO	NO	NO	NO	NO
rs11628933	14:60700903	C	-0.1696	PPM1A	YES	NO	NO	NO	NO	YES
rs11665020	18:10879503	C	-0.1205	PIEZO2	NO	NO	NO	NO	NO	NO
rs11853359	15:71621524	A	-0.1445	THSD4	NO	NO	NO	NO	NO	NO

rs11923667	3:101268080	A	0.1364	TRMT10C	NO	NO	NO	NO	NO	NO
rs12078697	1:117015118	C	-0.1102	RP4-655J12.4	YES	NO	NO	NO	YES	YES
rs12142296	1:46541679	T	-0.1959	PIK3R3	NO	NO	NO	NO	NO	NO
rs1220128	2:158499902	C	0.1161	ACVR1C	NO	NO	NO	NO	NO	NO
rs1232482	20:11886643	T	-0.0969	BTBD3	YES	NO	NO	NO	NO	YES
rs12374077	3:185317674	C	0.126	SENP2	YES	NO	NO	NO	YES	YES
rs12405515	1:172357441	T	-0.1617	PIGC	NO	NO	NO	NO	NO	NO
rs12408022	1:217718789	T	0.1486	GPATCH2	NO	NO	NO	NO	NO	NO
rs12486605	3:57706503	T	-0.122	DENND6A	YES	NO	NO	NO	NO	YES
rs12515541	5:57095011	T	0.0939	ACTBL2	NO	NO	NO	NO	NO	NO
rs12521868	5:131784393	T	-0.1463	C5orf56	NO	NO	NO	NO	NO	NO
rs12579720	12:20173764	C	-0.2713	RP11-664H17.1	NO	NO	NO	NO	NO	NO
rs12583615	13:50564085	A	0.1949	TRIM13	YES	NO	NO	NO	NO	YES
rs12627651	21:44760603	A	0.2479	SIK1	NO	NO	NO	NO	NO	NO
rs1271309	12:124820705	A	-0.1177	NCOR2	NO	NO	NO	NO	NO	NO
rs12906962	15:95312071	T	-0.1883	LOC440311	NO	NO	NO	NO	NO	NO
rs12921187	16:4943019	T	-0.1561	PPL	NO	NO	NO	NO	NO	NO
rs12940887	17:47402807	T	0.2476	ZNF652	YES	NO	NO	NO	NO	YES
rs13001283	2:127183454	A	0.1484	GYPC	NO	NO	NO	NO	NO	NO
rs13082711	3:27537909	T	-0.1835	SLC4A7	NO	NO	NO	NO	NO	NO
rs13107325	4:103188709	T	-0.6042	SLC39A8	YES	NO	NO	NO	NO	YES
rs13139571	4:156645513	A	-0.2307	GUCY1A3	NO	NO	NO	NO	NO	NO
rs13205180	6:51832494	T	0.1195	PKHD1	YES	NO	NO	NO	NO	YES
rs13209747	6:127115454	T	0.2542	NA	NO	NO	NO	NO	NO	NO
rs1327235	20:10969030	A	-0.239	RP11-103J8.1	NO	NO	NO	NO	NO	NO
rs13333226	16:20365654	A	0.253	UMOD	YES	NO	NO	NO	YES	YES
rs1378942	15:75077367	A	-0.3725	CSK	NO	NO	NO	NO	NO	NO
rs1438896	2:145646072	T	0.1912	TEX41	NO	NO	NO	NO	NO	NO
rs1458038	4:81164723	T	0.4107	FGF5	NO	NO	NO	NO	NO	NO
rs1530440	10:63524591	T	-0.3586	C10orf107	NO	NO	NO	NO	NO	NO
rs1565716	1:29549216	A	0.16	MECR	NO	NO	NO	NO	NO	NO
rs1607644	2:34679626	A	-0.1343	AC073218.1	NO	NO	NO	NO	NO	NO
rs167479	19:11526765	T	-0.3415	RGL3	YES	NO	NO	NO	NO	YES
rs16823124	2:183224127	A	0.1804	PDE1A	YES	NO	NO	NO	NO	YES
rs16851397	3:141134818	A	-0.4	ZBTB38	YES	NO	NO	NO	NO	YES
rs17030613	1:113190807	A	-0.2359	CAPZA1	YES	NO	NO	NO	NO	YES
rs1706003	3:194299967	T	0.1576	TMEM44-AS1	NO	NO	NO	NO	NO	NO
rs17080102	6:151004770	C	-0.5058	PLEKHG1	NO	NO	NO	NO	NO	NO
rs1718845	4:57943153	A	-0.0907	IGFBP7	NO	NO	NO	NO	NO	NO
rs17224476	11:4673788	A	0.1389	OR51E1	NO	NO	NO	NO	NO	NO
rs1722886	7:134215259	A	0.1009	AKR1B10	NO	NO	NO	NO	NO	NO
rs17248480	4:102435265	A	-0.4718	BANK1	NO	NO	NO	NO	NO	NO
rs17880989	14:23313633	A	0.2644	MMP14	NO	NO	NO	NO	NO	NO
rs1799945	6:26091179	C	-0.3791	HFE	NO	NO	NO	NO	NO	NO
rs1813353	10:18707448	T	0.2772	CACNB2	YES	NO	NO	NO	NO	YES
rs1821295	19:32590773	T	-0.1243	AC011518.2	NO	NO	NO	NO	NO	NO
rs1876487	2:73114352	A	-0.1105	SPR	NO	NO	NO	NO	NO	NO
rs1947228	7:96461649	T	-0.146	Metazoa_SRP	NO	NO	NO	NO	NO	NO
rs1975487	2:55809054	A	-0.1737	SMEK2	YES	NO	NO	NO	NO	YES
rs1996992	2:219651349	T	-0.2953	CYP27A1	NO	NO	NO	NO	NO	NO

rs2004776	1:230848702	T	0.2356	RP11-99J16__A.2	NO	NO	NO	YES	NO	YES
rs2034618	15:83799632	T	-0.1093	TM6SF1	YES	NO	NO	NO	NO	YES
rs2222544	7:69769369	T	-0.094	AUTS2	NO	NO	NO	NO	NO	NO
rs223361	4:103769304	T	0.0942	UBE2D3	YES	NO	NO	NO	YES	YES
rs2246438	10:45273079	A	-0.0714	TMEM72-AS1	NO	NO	NO	NO	NO	NO
rs2252865	1:8422676	T	0.0976	RERE	NO	NO	NO	NO	NO	NO
rs2304130	19:19789528	A	-0.2336	ZNF101	NO	NO	NO	NO	NO	NO
rs2306374	3:138119952	T	-0.1699	MRAS	NO	NO	NO	NO	NO	NO
rs2390258	2:166250129	A	-0.1132	SCN2A	NO	NO	NO	NO	NO	NO
rs2467099	17:73949045	T	-0.0837	ACOX1	NO	NO	NO	NO	NO	NO
rs2493292	1:3328659	T	0.1674	PRDM16	NO	NO	NO	NO	NO	NO
rs2521501	15:91437388	A	-0.3529	FES	NO	NO	NO	NO	NO	NO
rs2579519	2:96675166	T	-0.1499	RN7SL210P	NO	NO	NO	NO	NO	NO
rs2693560	6:117523671	A	-0.1391	VGLL2	NO	NO	NO	NO	NO	NO
rs2707238	2:38094149	C	0.0958	LINC00211	NO	NO	NO	NO	NO	NO
rs2782980	10:115781527	T	-0.2424	ADRB1	YES	NO	NO	NO	NO	YES
rs28590346	16:2080653	A	-0.1432	SLC9A3R2	NO	NO	NO	NO	NO	NO
rs28667801	4:26785356	A	-0.0908	TBC1D19	NO	NO	NO	NO	NO	NO
rs2925345	15:41311799	T	0.0706	INO80	YES	NO	NO	NO	YES	YES
rs2929184	11:6289118	A	0.1397	CCKBR	NO	NO	NO	NO	NO	NO
rs2932538	1:113216543	A	-0.237	MOV10	NO	NO	NO	NO	NO	NO
rs2969070	7:2512545	A	-0.198	GRIFIN	NO	NO	NO	NO	NO	NO
rs2978098	8:101676675	A	0.1223	SNX31	NO	NO	NO	NO	NO	NO
rs3184504	12:111884608	T	0.441	SH2B3	NO	NO	NO	NO	NO	NO
rs34163044	18:51851616	A	0.1494	STARD6	NO	NO	NO	NO	NO	NO
rs34324971	7:74107374	A	0.1341	AC083884.8	NO	NO	NO	NO	NO	NO
rs34517439	1:78450517	A	-0.165	DNAJB4	YES	NO	NO	NO	NO	YES
rs34570306	2:146272860	T	-0.0851	BC040861.1	NO	NO	NO	NO	NO	NO
rs34591516	8:142367087	T	0.2869	GPR20	NO	NO	NO	NO	NO	NO
rs35213536	20:62694319	T	0.1313	TCEA2	NO	YES	NO	NO	YES	YES
rs35444	12:115552437	A	0.2201	TBX3	NO	NO	NO	NO	NO	NO
rs35981664	1:218549354	A	-0.1327	TGFB2	YES	NO	NO	NO	NO	YES
rs36022378	3:49913705	T	-0.1144	ACTBP13	NO	NO	NO	NO	NO	NO
rs381815	11:16902268	T	0.2043	PLEKHA7	NO	NO	NO	NO	NO	NO
rs3822857	6:116313931	C	-0.105	FRK	YES	NO	NO	NO	NO	YES
rs3898618	12:120813921	T	-0.2039	RPS27P25	NO	NO	NO	NO	NO	NO
rs3918226	7:150690176	T	0.5454	NOS3	NO	NO	NO	NO	NO	NO
rs419076	3:169100886	T	0.25	MECOM	YES	NO	NO	NO	NO	YES
rs4245739	1:204518842	A	0.1364	MDM4	NO	NO	NO	NO	NO	NO
rs4286632	5:66291370	A	0.1548	MAST4	YES	NO	NO	NO	NO	YES
rs4292285	4:145271954	A	-0.099	GYPA	NO	NO	NO	NO	NO	NO
rs4308	17:61559625	A	0.1832	ACE	NO	NO	NO	YES	NO	YES
rs4364717	9:21801530	A	-0.0855	RP11-145E5.5	NO	NO	NO	NO	NO	NO
rs4373814	10:18419972	C	0.1595	CACNB2	YES	NO	NO	NO	NO	YES
rs4387287	10:105677897	A	0.1671	OBFC1	NO	NO	NO	NO	NO	NO
rs4411245	10:126712781	A	0.1317	CTBP2	NO	NO	NO	NO	NO	NO
rs4420291	11:74374950	A	0.0832	POLD3	NO	NO	NO	NO	NO	NO
rs4424827	14:35110857	T	-0.0849	SNX6	NO	NO	NO	NO	NO	NO
rs4494250	10:96563757	A	0.1767	CYP2C19	NO	NO	NO	NO	NO	NO
rs45474499	16:66914492	T	0.3021	PDP2	NO	NO	NO	NO	NO	NO

rs4634143	3:23163749	T	0.1024	UBE2E2	NO	NO	NO	NO	NO	NO
rs4714224	6:39186743	C	-0.1508	KCNK5	YES	NO	NO	NO	NO	YES
rs4757391	11:16302939	T	-0.2997	SOX6	NO	NO	NO	NO	NO	NO
rs4782211	16:19152219	A	0.105	CTD-2349B8.1	NO	NO	NO	NO	NO	NO
rs4800420	18:20158965	A	0.0884	CTAGE1	NO	NO	NO	NO	NO	NO
rs4810332	20:40268334	A	-0.1104	CHD6	YES	NO	NO	NO	NO	YES
rs4823006	22:29451671	A	0.1098	ZNRF3	NO	NO	NO	NO	NO	NO
rs4850047	2:3634753	T	-0.1402	RPS7	NO	NO	NO	NO	NO	NO
rs4851462	2:98357163	T	-0.0948	ZAP70	NO	NO	NO	NO	NO	NO
rs4903064	14:73279420	T	0.1164	DPF3	NO	NO	NO	NO	NO	NO
rs4924570	15:41974660	T	-0.1047	MGA	YES	NO	NO	NO	NO	YES
rs4952611	2:40567743	T	-0.1342	SLC8A1	NO	NO	NO	NO	NO	NO
rs4984496	15:96635898	T	0.1024	MIR1469	NO	NO	NO	NO	NO	NO
rs513177	12:69950545	T	0.1324	FRS2	YES	NO	NO	NO	NO	YES
rs55684003	13:97988689	A	0.111	MBNL2	YES	NO	NO	NO	NO	YES
rs55747751	5:132397351	A	-0.1912	HSPA4	YES	NO	NO	YES	NO	YES
rs55829085	4:2165493	A	-0.219	POLN	NO	NO	NO	NO	NO	NO
rs56233017	8:144981488	A	-0.2413	PLEC	NO	NO	NO	NO	NO	NO
rs56345595	8:82814156	A	0.0614	RP11-354A14.1	NO	NO	NO	NO	NO	NO
rs57927100	17:75317300	C	0.2378	40057	NO	NO	NO	NO	NO	NO
rs598682	6:154418759	A	-0.1398	OPRM1	NO	NO	NO	NO	NO	NO
rs6015450	20:57751117	A	-0.4801	NA	NO	NO	NO	NO	NO	NO
rs603424	10:102075479	A	0.1518	PKD2L1	NO	NO	NO	NO	NO	NO
rs6060114	20:30169673	T	0.1617	RNU6-384P	NO	NO	NO	NO	NO	NO
rs6095241	20:47308798	A	-0.1238	PREX1	NO	NO	NO	NO	NO	NO
rs6108168	20:8626271	A	-0.1415	PLCB1	NO	NO	NO	NO	NO	NO
rs61892344	11:101100768	T	-0.1129	PGR	YES	NO	NO	NO	NO	YES
rs61926181	12:50767037	A	-0.1357	FAM186A	NO	NO	NO	NO	NO	NO
rs62004794	15:68454523	A	-0.0905	PIAS1	YES	NO	NO	NO	NO	YES
rs62104477	19:30294991	T	0.1301	CCNE1	YES	NO	NO	NO	NO	YES
rs62229372	21:37692507	T	0.1568	MORC3	YES	NO	NO	NO	NO	YES
rs62503324	8:23400615	T	0.1635	SLC25A37	NO	NO	NO	NO	NO	NO
rs62524579	8:144060955	A	-0.1504	RP11-273G15.2	NO	NO	NO	YES	NO	YES
rs6271	9:136522274	T	-0.3647	DBH	NO	NO	NO	NO	NO	NO
rs633185	11:100593538	C	0.2799	ARHGAP42	NO	NO	NO	NO	NO	NO
rs6429422	1:243472801	T	-0.1919	SDCCAG8	NO	NO	NO	NO	NO	NO
rs6487543	12:26438189	A	0.1059	RP11-283G6.4	NO	NO	NO	NO	NO	NO
rs6565174	16:30111904	A	-0.1404	RP11-455F5.3	YES	NO	NO	NO	NO	YES
rs6681713	1:51527684	T	0.3834	MIR4421	NO	NO	NO	NO	NO	NO
rs668459	6:139835689	T	-0.1487	CITED2	NO	NO	NO	NO	NO	NO
rs6686889	1:25030470	T	0.115	SRRM1	YES	NO	NO	NO	NO	YES
rs66887589	4:120509279	T	-0.1251	PDE5A	YES	NO	NO	YES	NO	YES
rs67330701	11:69079707	T	-0.231	MYEOV	NO	NO	NO	NO	NO	NO
rs6777317	3:197070959	A	0.0855	snoU13	NO	NO	NO	NO	NO	NO
rs6797587	3:48197614	A	-0.2209	CDC25A	YES	NO	NO	NO	NO	YES
rs6825911	4:111381638	T	-0.1712	ENPEP	YES	NO	NO	NO	NO	YES
rs687621	9:136137065	A	0.1327	ABO	NO	NO	NO	NO	NO	NO
rs6891344	5:123136656	A	0.2182	CSNK1G3	YES	NO	NO	NO	NO	YES
rs7020564	9:109670016	A	-0.1083	ZNF462	NO	NO	NO	NO	NO	NO
rs7090758	10:65335315	T	-0.0755	REEP3	YES	NO	NO	NO	NO	YES

rs7103648	11:47461783	A	-0.1879	RAPSN	NO	NO	NO	NO	NO	NO
rs7132012	12:8832203	A	0.1229	RP11-20D14.4	NO	NO	NO	NO	NO	NO
rs7134060	12:96717095	A	-0.0816	CDK17	YES	NO	NO	NO	NO	YES
rs7137749	12:57098040	T	0.1039	NACA	NO	NO	NO	NO	NO	NO
rs7178615	15:66869072	A	-0.1328	hCG_2003567	NO	NO	NO	NO	NO	NO
rs7180952	15:85162551	T	-0.0911	ZSCAN2	YES	NO	NO	NO	NO	YES
rs7185555	16:69131281	C	-0.1528	HAS3	YES	NO	NO	NO	NO	YES
rs7215084	17:3880148	T	0.1443	RP11-459C13.1	NO	NO	NO	NO	NO	NO
rs72704264	1:145713305	C	0.1184	CD160	NO	NO	NO	NO	NO	NO
rs72799341	16:30936743	A	0.1572	FBXL19	YES	NO	NO	NO	NO	YES
rs72812846	5:173377636	A	-0.1698	CPEB4	NO	NO	NO	NO	NO	NO
rs72842207	10:121433675	T	-0.211	BAG3	NO	NO	NO	NO	NO	NO
rs7302981	12:50537815	A	0.2306	CERS5	NO	NO	NO	NO	NO	NO
rs73033340	7:1195692	A	0.2476	ZFAND2A	NO	NO	NO	NO	NO	NO
rs73049928	7:4669949	A	-0.1084	FOXK1	YES	NO	NO	NO	NO	YES
rs7313556	12:15297359	A	0.0973	RERG	NO	NO	NO	NO	NO	NO
rs745821	18:48142854	T	0.1411	MAPK4	NO	NO	NO	NO	NO	NO
rs7502046	17:19196440	T	0.1231	EPN2	YES	NO	NO	NO	NO	YES
rs751984	11:61278246	T	0.3632	LRRC10B	YES	NO	NO	NO	NO	YES
rs75507123	12:5417856	T	-0.1275	RP11-1038A11.3	NO	NO	NO	NO	NO	NO
rs7553422	1:119540719	T	-0.1164	RP4-712E4.2	YES	NO	NO	NO	NO	YES
rs7592578	2:191439591	T	-0.1875	7SK	NO	NO	NO	NO	NO	NO
rs7606205	2:144146311	A	-0.0994	AC096558.1	NO	NO	NO	NO	NO	NO
rs76326501	2:43167878	A	0.36	MIRN548C	NO	NO	NO	NO	NO	NO
rs76452347	9:35906471	T	-0.1691	HRCT1	NO	NO	NO	NO	NO	NO
rs7694000	4:95324968	A	-0.0921	PDLIM5	NO	NO	NO	NO	NO	NO
rs7694643	4:109017528	A	-0.0874	LEF1	NO	NO	NO	NO	NO	NO
rs79146658	2:179786068	T	-0.2819	CCDC141	NO	NO	NO	NO	NO	NO
rs8059962	16:81574197	T	-0.1138	CMIP	NO	NO	NO	NO	NO	NO
rs8069739	17:8078765	T	-0.137	TMEM107	NO	NO	NO	NO	NO	NO
rs873122	15:92702020	C	0.0954	SLCO3A1	NO	NO	NO	NO	NO	NO
rs875106	11:70005641	A	-0.0858	ANO1	NO	NO	NO	NO	NO	NO
rs882624	1:201735913	T	-0.1423	IPO9-AS1	NO	NO	NO	NO	NO	NO
rs891511	7:150704843	A	-0.2109	NOS3	NO	NO	NO	NO	NO	NO
rs894344	8:135612745	A	-0.0915	ZFAT	NO	NO	NO	NO	NO	NO
rs900145	11:13293905	T	0.1191	ARNTL	YES	NO	NO	NO	NO	YES
rs918466	3:64710253	A	-0.1637	ADAMTS9-AS2	NO	NO	NO	NO	NO	NO
rs9306160	21:45107562	T	-0.1687	RRP1B	YES	NO	NO	NO	NO	YES
rs9372498	6:118572486	A	0.1938	SLC35F1	NO	NO	NO	NO	NO	NO
rs9472135	6:43809802	T	0.1126	VEGFA	NO	NO	NO	NO	YES	YES
rs954767	5:3706050	A	-0.1074	IRX1	YES	NO	NO	NO	NO	YES
rs9563529	13:58316637	T	0.1014	PCDH17	NO	NO	NO	NO	NO	NO
rs9638084	7:156311745	A	0.0959	LINC01006	NO	NO	NO	NO	NO	NO
rs9687065	5:148391140	A	0.1789	SH3TC2	NO	NO	NO	NO	NO	NO
rs9810888	3:53635595	T	-0.128	CACNA1D	NO	NO	NO	NO	NO	NO
rs9827472	3:56726646	T	-0.1426	FAM208A	YES	NO	NO	NO	NO	YES
rs9865843	3:7489993	A	-0.0856	GRM7	NO	NO	NO	NO	NO	NO
rs9932866	16:706067	A	0.0935	LA16c-349E10.1	YES	NO	NO	NO	NO	YES

Table S2: Variance in blood pressure explained by genetic risk scores

Genetic instrument	Amount of variance explained by genetic instrument
**GRS using 219 SBP SNPs**	
R^2 from linear regression*	1.72%
Approx R^2 using summarised data†	1.60%
**GRS using 129 SBP SNPs excluding variants with potentially direct effects on the kidney‡**	
R^2 from linear regression*	0.95%
Approx R^2 using summarised data†	0.88%
**GRS using 223 DBP SNPs**	
R^2 from linear regression*	2.56%
Approx R^2 using summarised data†	2.11%
**GRS using 156 DBP SNPs excluding variants with potentially direct effects on the kidney‡**	
R^2 from linear regression*	1.82%
Approx R^2 using summarised data†	1.49%

GRS=genetic risk score. SBP=systolic blood pressure. DBP=diastolic blood pressure. *R^2 from linear regression of blood pressure on GRS using individual participant data from 311,119 individuals in UK Biobank. †Approximate R^2 using formulae from Burgess S, Dudbridge F, Thompson SG. Combining information on multiple instrumental variables in Mendelian randomization: comparison of allele score and summarized data methods. Stat Med. 2016 May 20;35(11):1880-906. ‡Variants identified through Steiger filtering as more strongly correlated with estimated glomerular filtration rate or urinary albumin:creatinine ratio than blood pressure, or in genes differentially expressed in the kidney, or associated with TGF-beta signalling, the renin-angiotensin system or disordered kidney development, morphology or physiology, were excluded from the genetic risk scores.

Table S3: Cross tabulation of eGFR and uACR categories in UK Biobank

	uACR category				
eGFR category	Undetectable	uACR <3 mg/mmol	uACR ≥3 to <30 mg/mmol	uACR ≥30 mg/mmol	All participants
eGFR ≥120 ml/min/1.73m²	1329 (0.4%)	382 (0.1%)	103 (0.0%)	14 (0.0%)	1828 (0.6%)
eGFR ≥90 to <120 ml/min/1.73m²	117016 (37.6%)	37872 (12.2%)	6244 (2.0%)	336 (0.1%)	161468 (51.9%)
eGFR ≥60 to <90 ml/min/1.73m²	93316 (30.0%)	39581 (12.7%)	6648 (2.1%)	497 (0.2%)	140042 (45.0%)
eGFR ≥45 to <60 ml/min/1.73m²	2887 (0.9%)	2149 (0.7%)	744 (0.2%)	156 (0.1%)	5936 (1.9%)
KRT or eGFR <45 ml/min/1.73m²	563 (0.2%)	524 (0.2%)	471 (0.2%)	287 (0.1%)	1845 (0.6%)
All participants	215111 (69.1%)	80508 (25.9%)	14210 (4.6%)	1290 (0.4%)	311119 (100%)

uACR=urinary albumin:creatinine ratio. eGFR=estimated glomerular filtration rate. KRT=kidney replacement therapy. Restricted to 311,119 genotyped white British participants (with related participants excluded).

Figure S1: Differential expression analysis of BP genes across GTEx v7 53 tissue types.

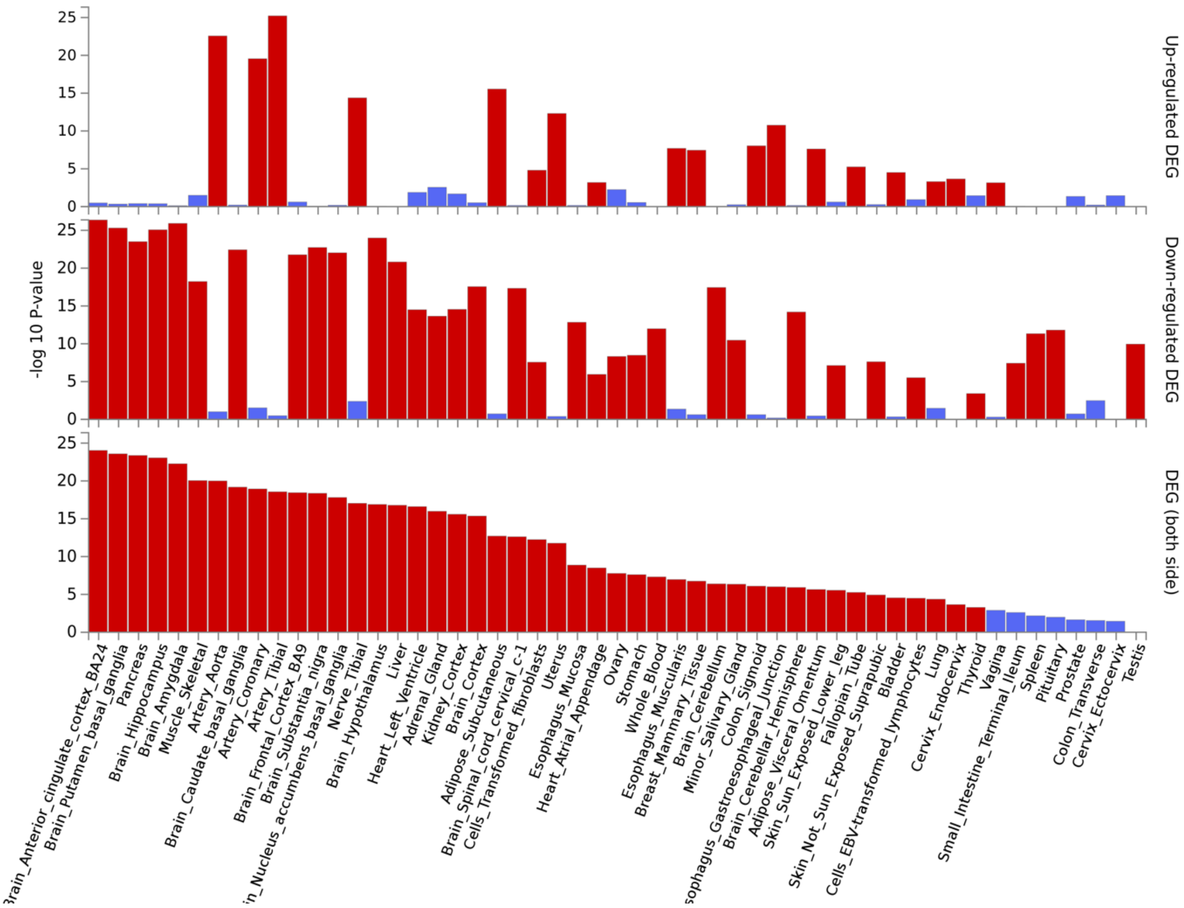

Significant enrichment at Bonferroni corrected P ≤ 0.05 are coloured in red.

Figure S2: Distributions of genetic risk scores for systolic and diastolic blood pressure

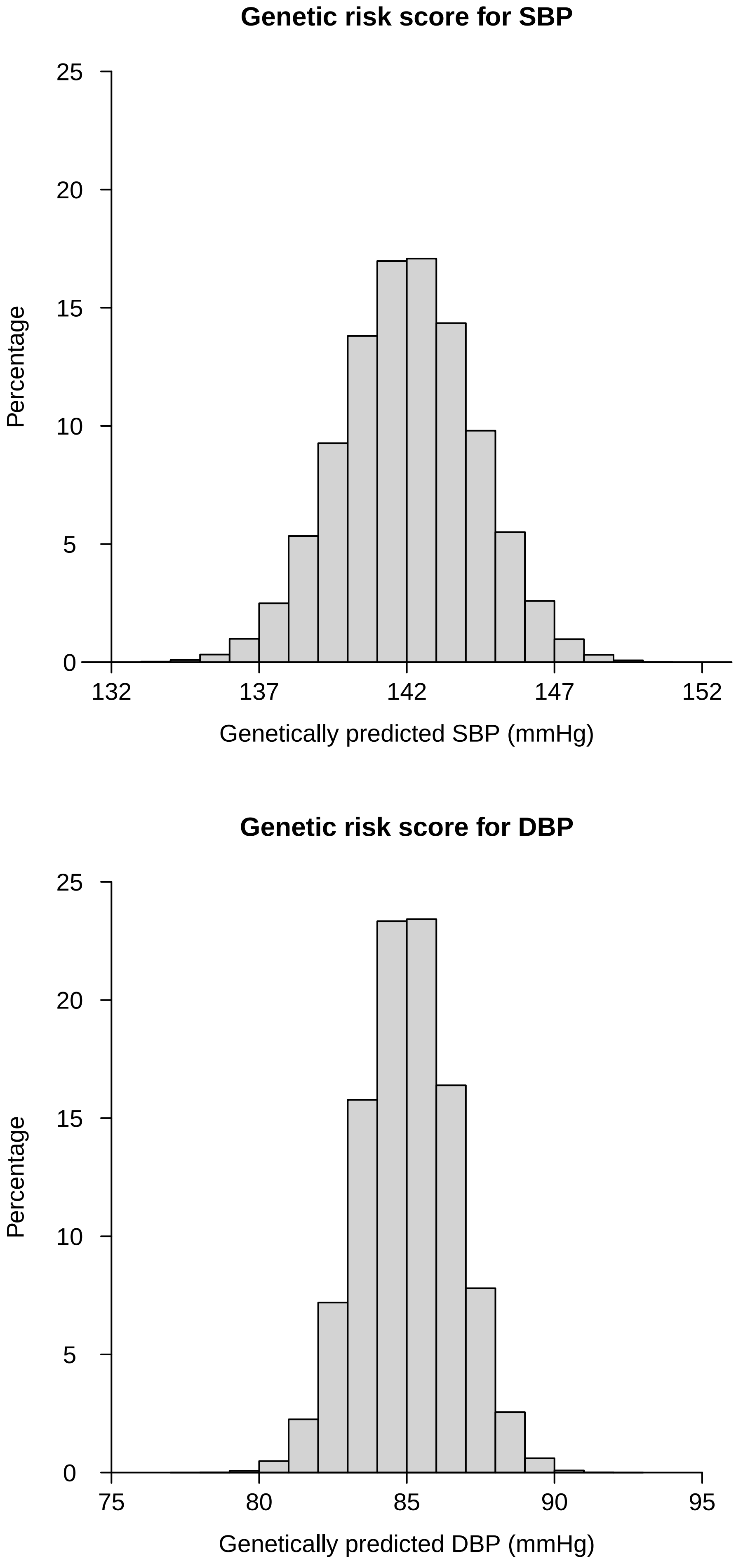

Figure S3: Association of genetically predicted blood pressure with estimated glomerular filtration rate and urinary albumin:creatinine ratio categories

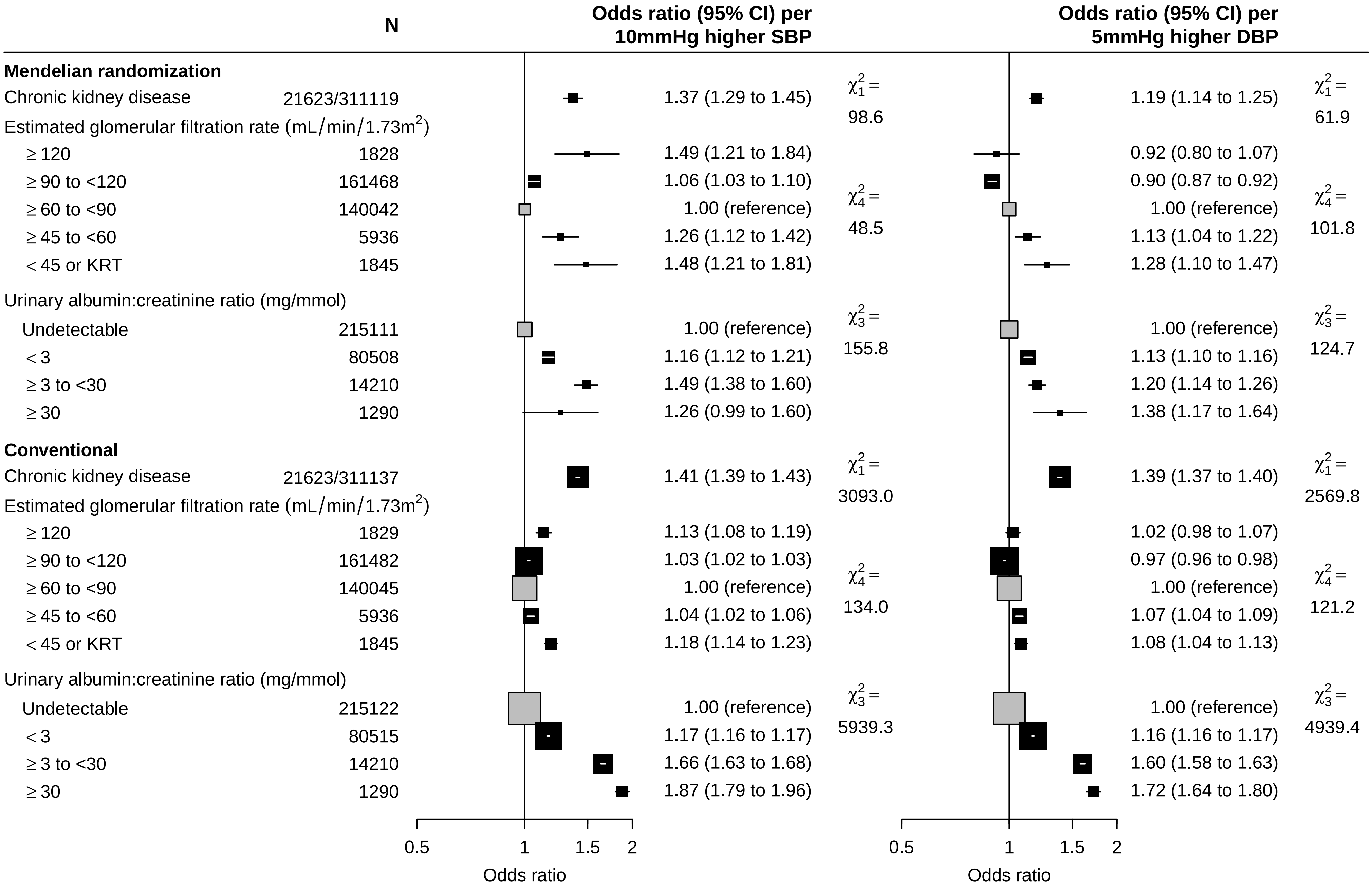

Chronic kidney disease defined as long−term kidney replacement therapy, estimated glomerular filtration rate <60mL/min/1.73m^2 or urinary albumin:creatinine ratio ≥ 3mg/mmol. KRT=kidney replacement therapy. SBP=systolic blood pressure. DBP=diastolic blood pressure. Mendelian randomization analyses adjusted for age, age², sex, measured body mass index, top 18 principal components and array. Conventional analyses adjusted for age, sex, ethnicity, education, region, deprivation index, smoking status, drinking status, physical activity, measured body mass index and diabetes status. The size of boxes is proportional to the amount of information in that category and boxes for reference categories are coloured grey.

Figure S4: Association of genetically predicted blood pressure with chronic kidney disease, by residuals of selected characteristics

	n/N	Odds ratio (95% CI) per 10mmHg higher SBP	p value for het/ trend test	n/N	Odds ratio (95% CI) per 5mmHg higher DBP	p value for het/ trend test
**HbA1c (%)***						
< 5.7	13406/242719	1.36 (1.26 to 1.47)	0.29	13414/242754	1.20 (1.13 to 1.26)	0.64
≥ 5.7	3846/38730	1.24 (1.07 to 1.44)		3838/38695	1.23 (1.11 to 1.37)	
**10 year cardiovascular risk (%)†**						
< 5	6102/161531	1.27 (1.14 to 1.43)	0.72	6089/161537	1.19 (1.09 to 1.29)	0.22
≥ 5 to <10	3960/53816	1.36 (1.18 to 1.57)		3977/53820	1.23 (1.11 to 1.36)	
≥ 10	6390/47502	1.31 (1.17 to 1.47)		6386/47492	1.10 (1.02 to 1.20)	
**Body mass index (kg/m²)**						
< 25	5134/103296	1.47 (1.30 to 1.66)	0.19	5161/103273	1.18 (1.08 to 1.29)	0.98
≥ 25 to <30	8227/133485	1.36 (1.23 to 1.50)		8197/133540	1.21 (1.13 to 1.30)	
≥ 30	8262/74338	1.32 (1.19 to 1.46)		8265/74306	1.18 (1.10 to 1.27)	
**Waist−to−hip ratio**						
< 0.83	5492/107022	1.34 (1.19 to 1.51)	0.53	5495/107041	1.18 (1.08 to 1.28)	0.54
≥ 0.83 to <0.92	6631/107022	1.34 (1.20 to 1.50)		6618/107020	1.25 (1.16 to 1.36)	
≥ 0.92	9482/97002	1.40 (1.28 to 1.54)		9492/96985	1.15 (1.08 to 1.24)	
**All participants**	**21623/311119**	**1.37 (1.29 to 1.45)**		**21623/311119**	**1.19 (1.14 to 1.25)**	

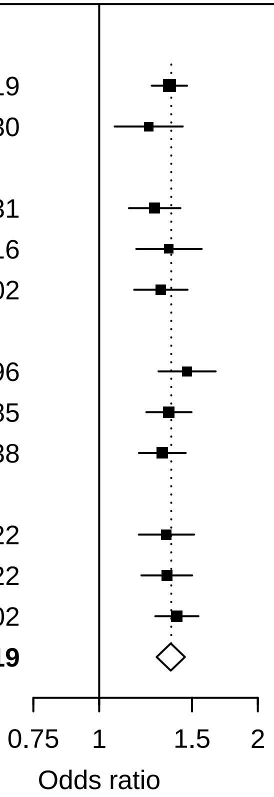

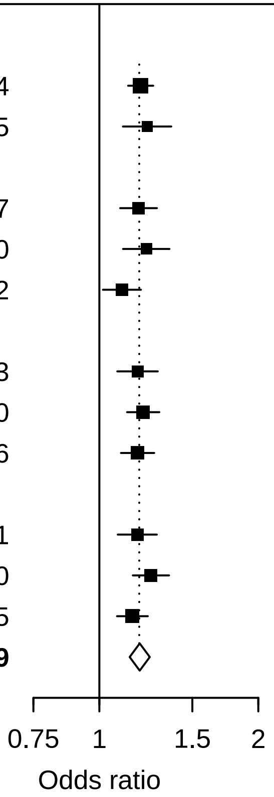

Chronic kidney disease defined as long−term kidney replacement therapy, estimated glomerular filtration rate <60mL/min/1.73m^2 or urinary albumin:creatinine ratio ≥ 3mg/mmol. SBP=systolic blood pressure. DBP=diastolic blood pressure. het=heterogeneity. Analyses adjusted for age, age², sex, measured body mass index, top 18 principal components and array. Residual baseline characteristics (defined as the participant's value of the characteristic minus the genetic contribution to the characteristic from the BP GRS) were calculated as the residual from regression of the baseline characteristic on the genetic risk scores for blood pressure. *Excludes participants with known diabetes. †10 year cardiovascular risk estimated using QRISK3 and analyses restricted to participants without prior cardiovascular disease at baseline.

Figure S5: Association of genetically predicted blood pressure with estimated glomerular filtration rate and urinary albumin:creatinine ratio categories, after excluding 90 and 67 variants

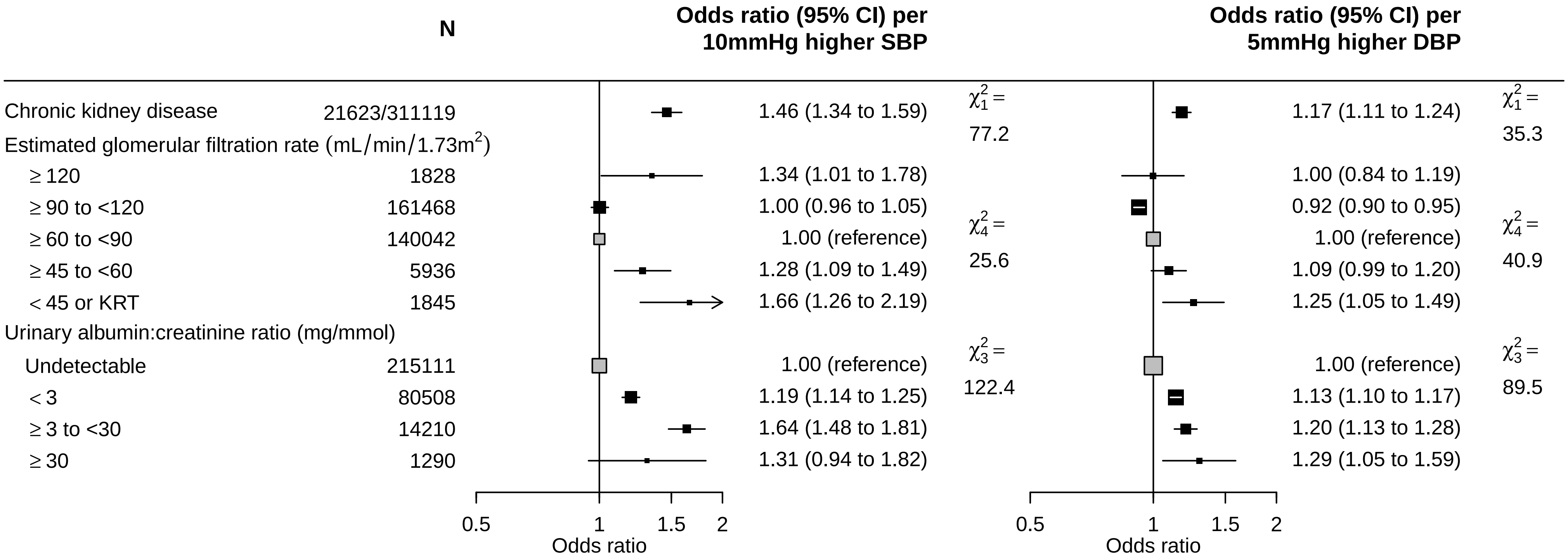

Chronic kidney disease defined as long−term kidney replacement therapy, estimated glomerular filtration rate <60mL/min/1.73m^2 or urinary albumin:creatinine ratio ≥ 3mg/mmol. KRT=kidney replacement therapy. SBP=systolic blood pressure. DBP=diastolic blood pressure. Variants identified through Steiger filtering that explain more variation in estimated glomerular filtration rate or urinary albumin:creatinine ratio than blood pressure (assuming no measurement error), or in genes differentially expressed in the kidney, or associated with TGF−beta signalling, the renin−angiotensin system or disordered kidney development, morphology or physiology, were excluded from the genetic risk scores. Mendelian randomization analyses adjusted for age, age², sex, measured body mass index, top 18 principal components and array. The size of boxes is proportional to the amount of information in that category and boxes for reference categories are coloured grey.

Figure S6: Association of genetically predicted blood pressure with chronic kidney disease and hyperfiltration in two−sample summary data Mendelian randomisation analyses

	Intercept for MR Egger for SBP	Odds ratio (95% CI) per 10mmHg higher SBP	Intercept for MR Egger for DBP	Odds ratio (95% CI) per 5mmHg higher DBP
**Chronic kidney disease**				
Inverse−variance weighted (IVW)		1.38 (1.30, 1.47)		1.20 (1.15, 1.25)
MR−Egger	1.004 (1.001, 1.007)	1.20 (1.05, 1.37)	0.999 (0.996, 1.003)	1.22 (1.10, 1.35)
Weighted median		1.30 (1.17, 1.45)		1.21 (1.12, 1.31)
Outlier corrected IVW (Modified Q statistic)		1.34 (1.26, 1.43)		1.18 (1.13, 1.23)
Outlier corrected IVW (MR−PRESSO)		1.34 (1.24, 1.44)		1.16 (1.10, 1.23)
**Hyperfiltration**				
Inverse−variance weighted (IVW)		1.55 (1.23, 1.96)		0.90 (0.76, 1.06)
MR−Egger	1.006 (0.993, 1.018)	1.27 (0.78, 2.08)	1.002 (0.989, 1.015)	0.86 (0.59, 1.24)
Weighted median		1.34 (0.91, 1.98)		0.97 (0.73, 1.27)
Outlier corrected IVW (Modified Q statistic)		1.44 (1.14, 1.81)		0.99 (0.84, 1.17)
Outlier corrected IVW (MR−PRESSO)		1.47 (1.11, 1.95)		0.97 (0.79, 1.21)

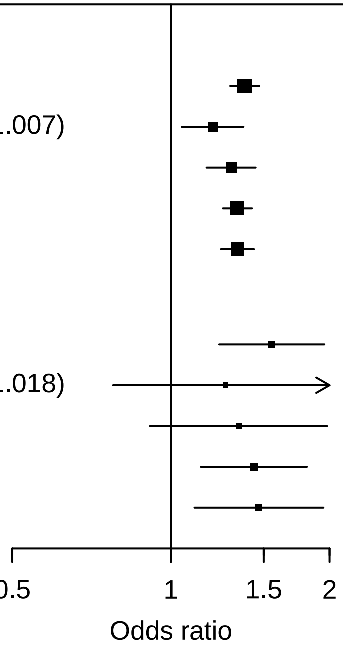

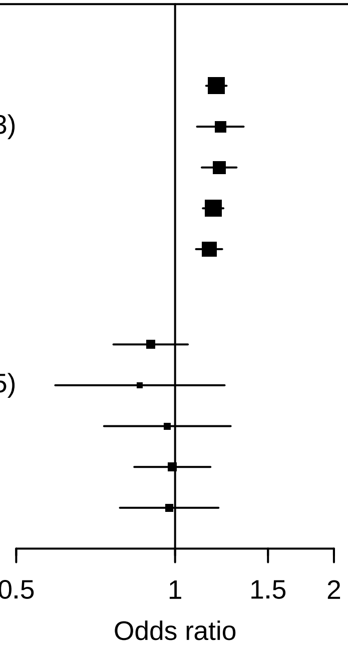

Chronic kidney disease defined as long−term kidney replacement therapy, estimated glomerular filtration rate <60mL/min/1.73m^2 or urinary albumin:creatinine ratio ≥ 3mg/mmol. Hyperfiltration defined as estimated glomerular filtration rate ≥ 120mL/min/1.73m^2. SBP=systolic blood pressure. DBP=diastolic blood pressure. An intercept significantly different to 1.00 for MR−Egger suggests the presence of directional pleiotropy.

Figure S7: Association of genetically predicted blood pressure with estimated glomerular filtration rate and urinary albumin:creatinine ratio categories, after adjustment for type 2 diabetes, body mass index and waist−to−hip ratio

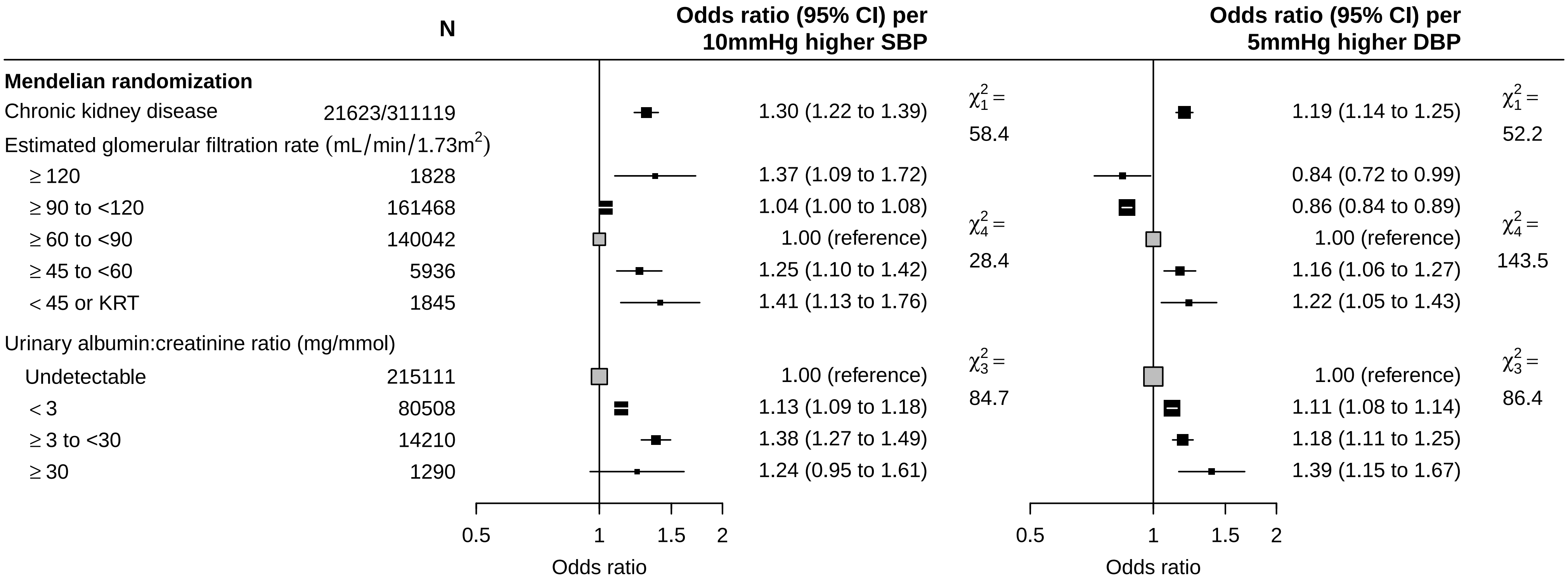

Chronic kidney disease defined as long−term kidney replacement therapy, estimated glomerular filtration rate <60mL/min/1.73m^2 or urinary albumin:creatinine ratio ≥ 3mg/mmol. KRT=kidney replacement therapy. SBP=systolic blood pressure. DBP=diastolic blood pressure. Mendelian randomization analyses adjusted for age, age², sex, measured body mass index, top 18 principal components, array and the genetic effect of the blood pressure SNPs on diabetes, body mass index and waist−to−hip ratio. The size of boxes is proportional to the amount of information in that category and boxes for reference categories are coloured grey.

Figure S8: Association of genetically predicted blood pressure with hyperfiltration, by selected characteristics

	n/N	Odds ratio (95% CI) per 10mmHg higher SBP	p value for het/ trend test	Odds ratio (95% CI) per 5mmHg higher DBP	p value for het/ trend test
**Age (years)**					
< 50	1505/12945	1.58 (1.25 to 2.01)	0.53	0.94 (0.79 to 1.11)	0.97
≥ 50 to <60	280/40693	1.07 (0.64 to 1.80)		0.87 (0.60 to 1.25)	
≥ 60	43/88232	2.39 (0.64 to 8.96)		1.13 (0.44 to 2.88)	
**Sex**					
Female	1204/74020	1.49 (1.15 to 1.93)	0.92	0.91 (0.76 to 1.09)	0.75
Male	624/67850	1.52 (1.07 to 2.17)		0.96 (0.75 to 1.23)	
**Prior diabetes**					
No	1699/133695	1.62 (1.31 to 2.01)	7e−04	0.93 (0.80 to 1.08)	0.41
Yes	129/8175	0.39 (0.18 to 0.86)		0.72 (0.40 to 1.29)	
**Prior vascular disease**					
No	1794/130809	1.49 (1.21 to 1.84)	0.81	0.93 (0.80 to 1.07)	0.85
Yes	34/11061	1.79 (0.39 to 8.21)		0.84 (0.30 to 2.38)	
**Body mass index (kg/m²)**					
< 25	990/36410	1.26 (0.95 to 1.68)	0.24	1.00 (0.82 to 1.23)	0.09
≥ 25 to <30	599/63681	1.98 (1.39 to 2.84)		0.92 (0.71 to 1.18)	
≥ 30	239/41779	1.46 (0.83 to 2.57)		0.66 (0.44 to 0.99)	
**Waist−to−hip ratio**					
< 0.83	923/41674	1.27 (0.95 to 1.71)	0.22	1.01 (0.82 to 1.24)	0.56
≥ 0.83 to <0.92	592/48844	1.84 (1.28 to 2.64)		0.79 (0.61 to 1.02)	
≥ 0.92	313/51352	1.65 (1.00 to 2.70)		0.97 (0.69 to 1.38)	
**All participants**	**1828/141870**	**1.49 (1.21 to 1.84)**		**0.92 (0.80 to 1.07)**	

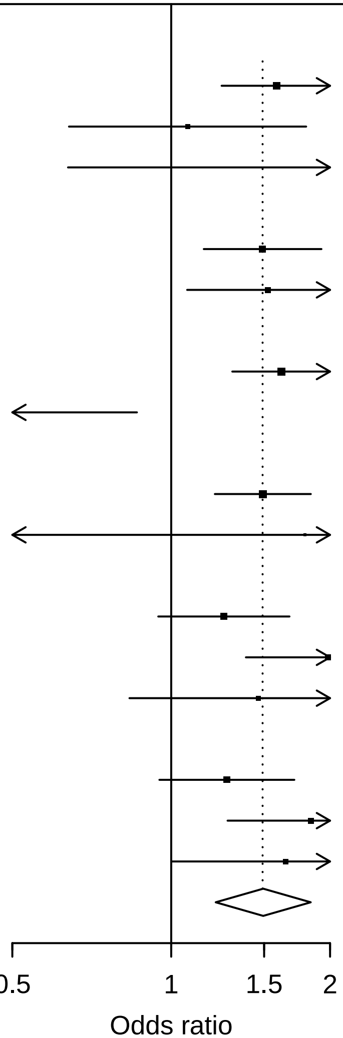

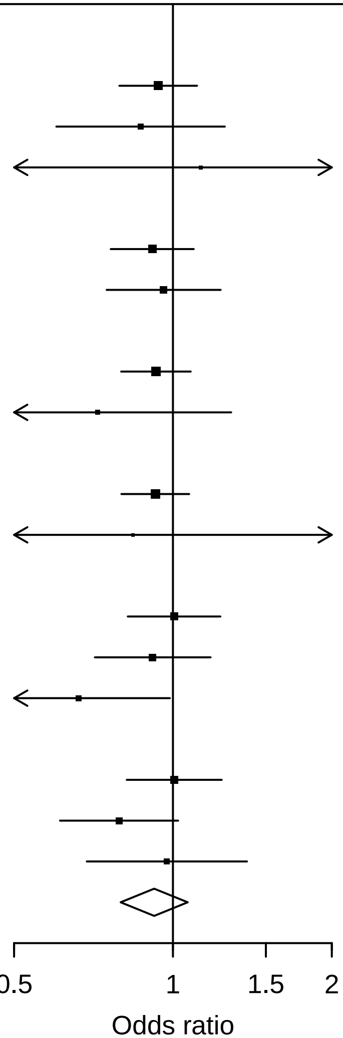

Hyperfiltration defined as estimated glomerular filtration rate ≥120mL/min/1.73m^2. SBP=systolic blood pressure. DBP=diastolic blood pressure. Analyses adjusted for age, age², sex, measured body mass index, top 18 principal components and array.

Figure S9: Association of blood pressure with acute kidney injury

	Odds ratio (95% CI) per 10mmHg higher SBP	χ^2_1	Odds ratio (95% CI) per 5mmHg higher DBP	χ^2_1
**Mendelian randomization**				
Basic adjustment*	1.27 (1.16 to 1.39)	28.0	1.09 (1.02 to 1.16)	6.6
+ eGFR and number of hospitalizations	1.20 (1.10 to 1.32)	15.4	1.00 (0.94 to 1.07)	7e−04
+ diabetes	1.15 (1.04 to 1.27)	7.2	1.00 (0.94 to 1.07)	7e−04
+ adiposity	1.15 (1.04 to 1.28)	7.5	1.01 (0.94 to 1.09)	0.1
**Conventional**				
Confounder adjusted†	1.23 (1.21 to 1.25)	574.4	1.19 (1.17 to 1.21)	404.9
+ eGFR and number of hospitalizations	1.21 (1.19 to 1.23)	453.1	1.14 (1.12 to 1.16)	213.5
+ diabetes	1.19 (1.17 to 1.21)	370.3	1.14 (1.12 to 1.16)	202.2
+ adiposity	1.18 (1.16 to 1.20)	310.1	1.12 (1.10 to 1.14)	134.3

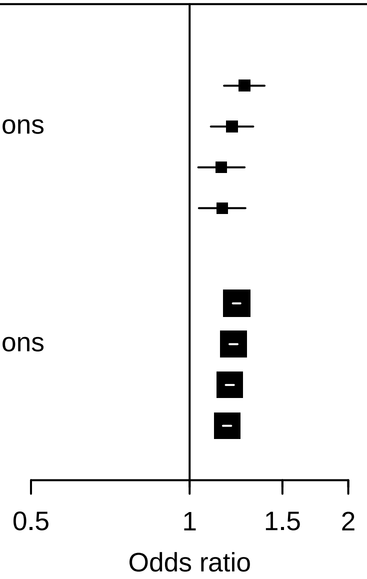

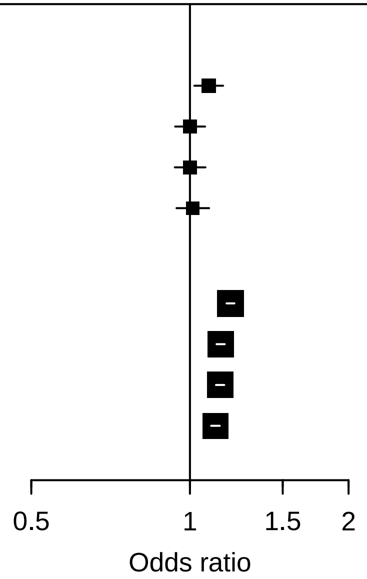

SBP=systolic blood pressure. DBP=diastolic blood pressure. Analyses included 311,119 participants with 10,122 cases of acute kidney injury. *Mendelian randomization analyses adjusted for age, age², sex, BMI, top 18 principal components and array. Multivariable Mendelian randomization analyses also adjusted for genetic effects of diabetes, body mass index and waist−to−hip ratio. †Conventional analyses adjusted for age, sex, ethnicity, education, region, deprivation index, smoking status, drinking status and physical activity.

Figure S10: Association of genetically predicted blood pressure with acute kidney injury, by selected characteristics

	n/N	Odds ratio (95% CI) per 10mmHg higher SBP	p value for het/ trend test	Odds ratio (95% CI) per 5mmHg higher DBP	p value for het/ trend test
**Age (years)**					
< 50	588/68281	1.45 (1.02 to 2.07)	0.58	0.92 (0.71 to 1.18)	0.08
≥ 50 to <60	2193/102824	1.10 (0.92 to 1.33)		1.02 (0.90 to 1.17)	
≥ 60	7341/140014	1.31 (1.18 to 1.46)		1.12 (1.04 to 1.21)	
**Sex**					
Female	3762/166452	1.20 (1.04 to 1.38)	0.32	1.01 (0.92 to 1.12)	0.1
Male	6360/144667	1.32 (1.18 to 1.47)		1.13 (1.04 to 1.23)	
**Prior diabetes**					
No	8086/294837	1.20 (1.09 to 1.33)	0.43	1.08 (1.01 to 1.16)	0.47
Yes	2036/16282	1.32 (1.07 to 1.63)		1.02 (0.88 to 1.18)	
**Prior vascular disease**					
No	8152/292951	1.21 (1.10 to 1.34)	0.47	1.06 (0.99 to 1.13)	0.61
Yes	1970/18168	1.32 (1.07 to 1.63)		1.01 (0.87 to 1.18)	
**Body mass index (kg/m²)**					
< 25	2028/103347	1.25 (1.03 to 1.52)	0.23	1.03 (0.90 to 1.19)	0.38
≥ 25 to <30	3977/133405	1.17 (1.02 to 1.35)		1.08 (0.98 to 1.19)	
≥ 30	4117/74367	1.39 (1.21 to 1.60)		1.12 (1.01 to 1.24)	
**Waist−to−hip ratio**					
< 0.83	1607/106814	1.29 (1.04 to 1.60)	0.17	0.89 (0.77 to 1.04)	0.02
≥ 0.83 to <0.92	2967/106932	1.07 (0.91 to 1.25)		1.11 (0.98 to 1.24)	
≥ 0.92	5548/97373	1.39 (1.23 to 1.56)		1.13 (1.04 to 1.23)	
**Estimated glomerular filtration rate**					
< 60 or KRT	1711/7781	1.11 (0.87 to 1.41)	0.28	1.05 (0.88 to 1.25)	0.08
≥ 60 to <90	5863/140042	1.22 (1.09 to 1.38)		1.10 (1.01 to 1.19)	
≥ 90	2548/163296	1.34 (1.13 to 1.59)		0.97 (0.86 to 1.10)	
**All participants**	**10122/311119**	**1.27 (1.16 to 1.39)**		**1.09 (1.02 to 1.16)**	

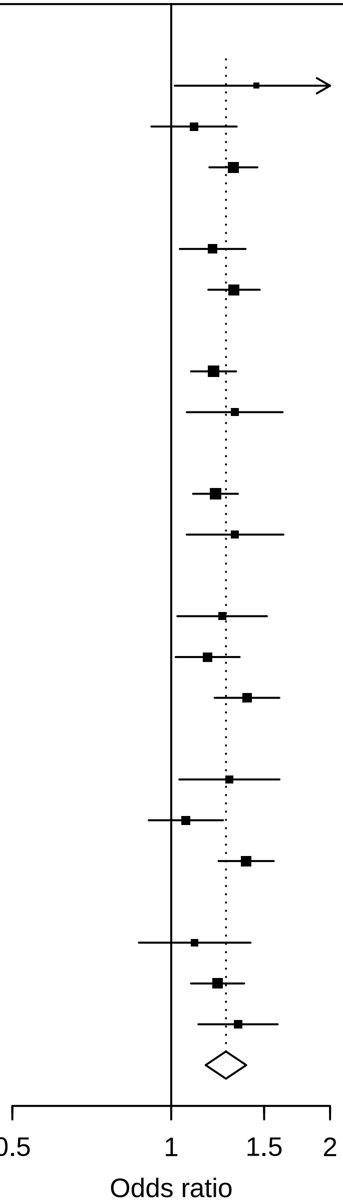

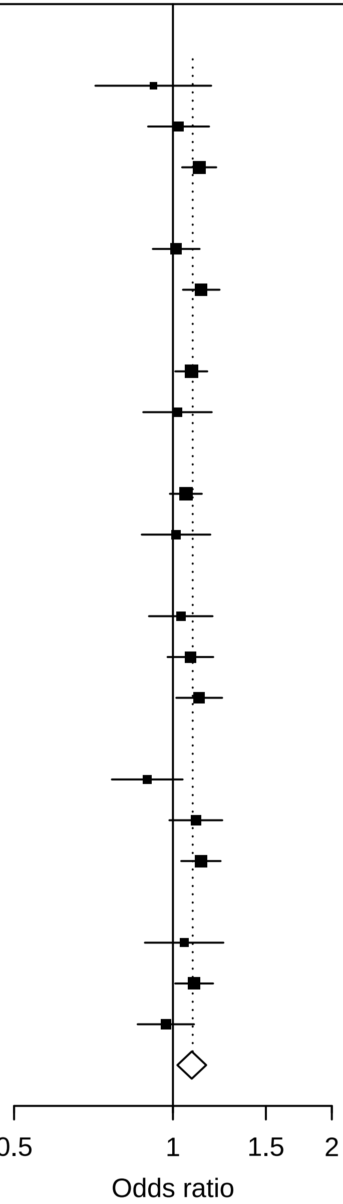

SBP=systolic blood pressure. DBP=diastolic blood pressure. Analyses adjusted for age, age², sex, measured body mass index, top 18 principal components and array.

Figure S11: Association of measured blood pressure with (a) estimated glomerular filtration rate and (b) urinary albumin:creatinine ratio categories

(a) Estimated glomerular filtration rate

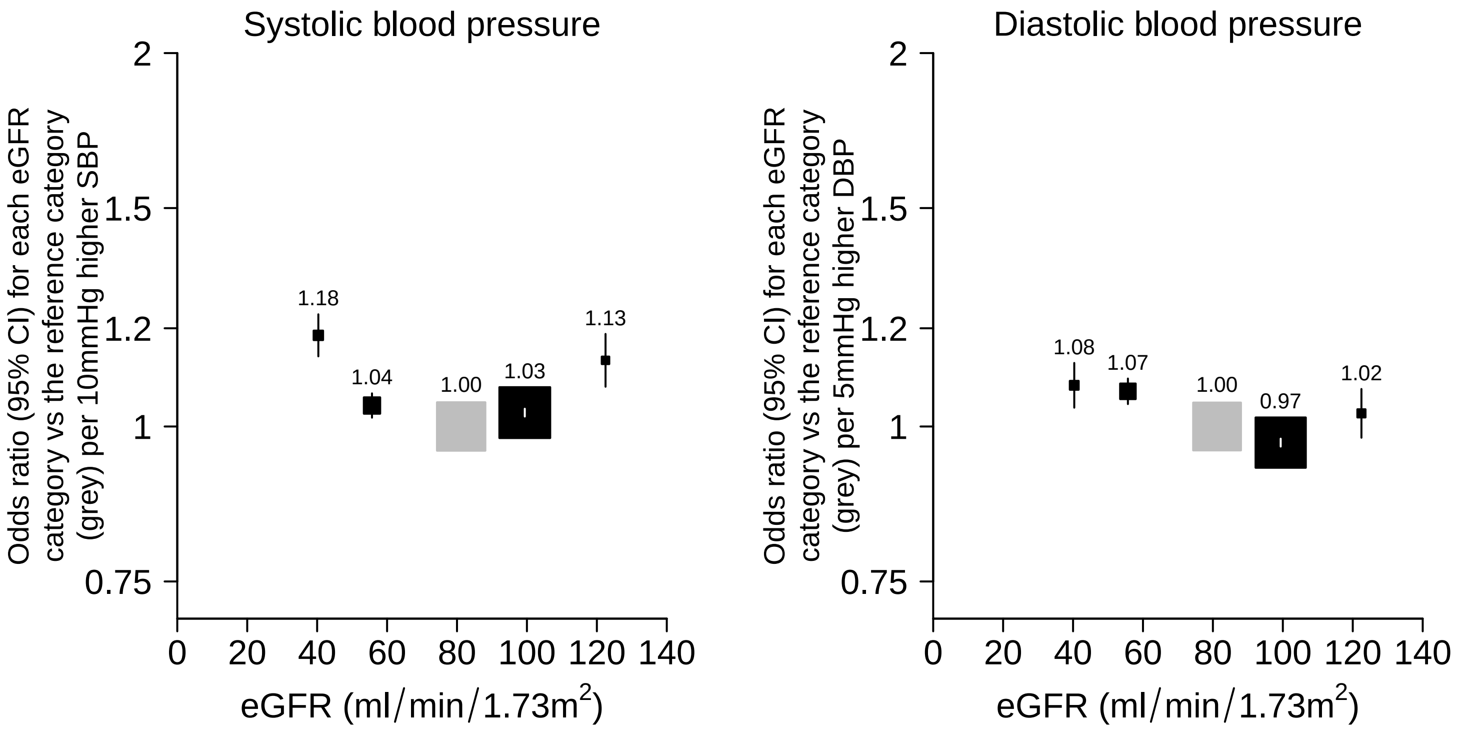

(b) Urinary albumin:creatinine ratio

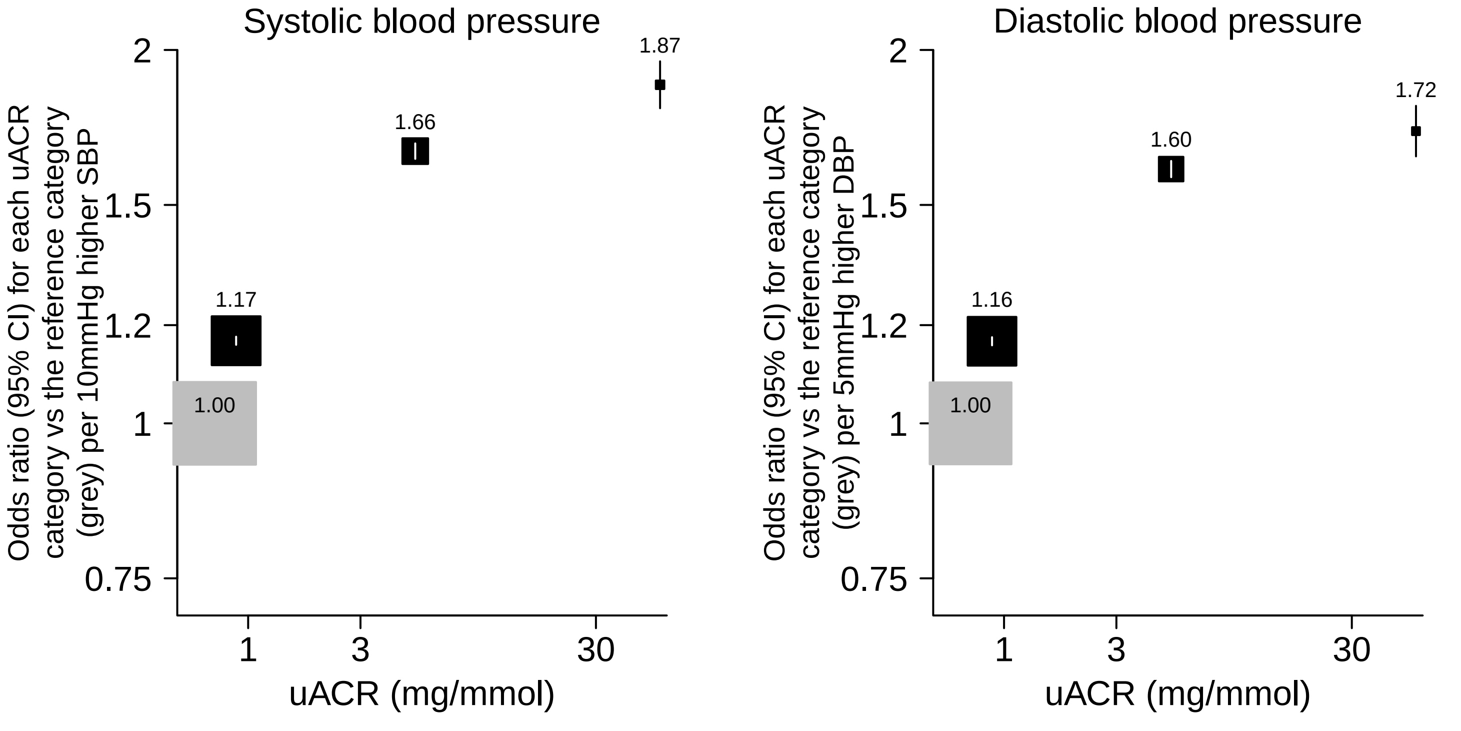

SBP=systolic blood pressure. DBP=diastolic blood pressure. eGFR=estimated glomerular filtration rate. uACR=urinary albumin:creatinine ratio. Grey boxes indicate reference categories. Analyses adjusted for age, age², sex, measured body mass index, top 18 principal components and array.
